# Accurate lattice parameters from 3D electron diffraction data. I. Optical distortions

**DOI:** 10.1107/S2052252522007904

**Published:** 2022-09-27

**Authors:** Petr Brázda, Mariana Klementová, Yaşar Krysiak, Lukáš Palatinus

**Affiliations:** a Department of Structure Analysis, Institute of Physics of the Czech Academy of Sciences, Na Slovance 1999/2, Prague 8, 18221, Czech Republic; b Institute of Inorganic Chemistry, Leibniz University of Hannover, Callinstraße 9, Hannover, 30167, Germany

**Keywords:** 3D electron diffraction, distortions, lattice parameters, parabolic distortion, precession electron diffraction

## Abstract

Determination of lattice parameters from 3D electron diffraction (3D ED) data measured in a transmission electron microscope is hampered by a number of effects that seriously limit the achievable accuracy. The distortion of the diffraction patterns by the optical elements of the microscope is often the most severe problem. A thorough analysis of a number of experimental datasets shows that, in addition to the well known distortions, namely barrel-pincushion, spiral and elliptical, an additional distortion, dubbed parabolic, may be observed in the data. In precession electron diffraction data, the parabolic distortion leads to excitation-error-dependent shift and splitting of reflections. All distortions except for the elliptical distortion can be determined together with lattice parameters from a single 3D ED data set. However, the parameters of the elliptical distortion cannot be determined uniquely due to correlations with the lattice parameters. They can be determined and corrected either by making use of the known Laue class of the crystal or by combining data from two or more crystals. The 3D ED data can yield lattice parameter ratios with an accuracy of about 0.1% and angles with an accuracy better than 0.03°.

## Abstract 2

Refinement of optical distortions in 3D electron diffraction data allows the determination of accurate lattice parameters and improves intensity integration.

## 1. Introduction

Three-dimensional electron diffraction (3D ED) (Kolb *et al.*, 2007 ▸; Zhang *et al.*, 2010 ▸) has been undergoing rapid development in recent years (Gemmi *et al.*, 2019 ▸). Structure solution is relatively easy, and dynamic refinement provides accurate structure models (Palatinus *et al.*, 2017 ▸) and also enables absolute structure determination (Brázda *et al.*, 2019 ▸). However, the accuracy of the lattice parameters remains low, with an order of magnitude or lower accuracy than single-crystal X-ray data, and even worse than that compared with powder X-ray data. The reasons for this poor accuracy are the omission of the fact that magnification is dependent on lens excitation, instrument-induced geometric distortions present in the data, mechanical instabilities of the microscope goniometer and, in the case of beam-sensitive samples, crystal structure changes induced by electron beam damage (see the example for borane at http://pets.fzu.cz/). In 2D diffraction patterns, elliptical, barrel-pincushion and spiral distortions caused by aberrations of electromagnetic lenses are well known and have been analysed several times (Hall, 1966 ▸; Williams & Carter, 2009 ▸; Capitani *et al.*, 2006 ▸; Mugnaioli *et al.*, 2009 ▸; Mitchell & Van den Berg, 2016 ▸; Palatinus *et al.*, 2019 ▸; Bücker *et al.*, 2021 ▸). Hüe *et al.* (2005 ▸) analysed the geometric distortions in images. Disparate attempts to address some of the distortion problems connected with the 3D reconstruction of reciprocal space can be found (Kolb *et al.*, 2008 ▸; Smeets *et al.*, 2018 ▸; Clabbers *et al.*, 2018 ▸; Ångström *et al.*, 2018 ▸; Mahr *et al.*, 2019 ▸). However, a thorough and deep analysis of the distortions in 3D ED data and their correlation with and impact on the lattice parameters has not yet been reported.

The effects leading to inaccurate lattice parameters can be divided into three main categories:

(i) Optical distortions: distortions of the diffraction pattern introduced by the optics of the electron microscope.

(ii) Mechanical instabilities: distortions of the diffraction pattern induced by the imperfect mechanics of the microscope, mainly the goniometer, and possibly by the sample properties, *e.g.* movement of the crystal on the support membrane during the experiment.

(iii) Radiation damage: in this case the lattice parameters of a beam-sensitive material often expand during the experiment, which induces an apparent change in magnification.

The effect of the distortions is twofold. First, the determined unit-cell geometry may be strongly distorted, with severe consequences ranging from problems with identification of the phase in databases or an inability to distinguish different phases having similar lattice parameters simultaneously present in the material, through problems with the determination of the crystal system, to the simple fact that one of the crucial results of structure determination – the unit-cell parameters – are not well determined. Second, the low accuracy of the predicted positions of the reflections on the diffraction patterns leads to problems with intensity integration.

In three consecutive publications, we will analyse all these effects and provide a pathway to accurate lattice parameter determination from 3D ED data, as well as an assessment of the limitations on the achievable accuracy. In this paper (Part I), we focus on the effects of the microscope optics on the distortions of the diffraction patterns and thus also on the determination of the lattice parameters. We assume that the optical distortions are constant within a given dataset. We analyse the distortions in static-beam geometry and precession-assisted diffraction patterns. When the distortions do not change within a dataset, the static-beam geometry is then also an accurate model for the continuous-rotation geometry (Nederlof *et al.*, 2013 ▸; Nannenga *et al.*, 2014 ▸), which is nowadays the most popular one for data acquisition. In the next paper (Part II; Brázda & Palatinus, 2023*a* ▸), we will discuss the effect of mechanical instabilities, changes in geometry during the experiment, which inevitably bring about changes in distortions, and the effect of radiation damage. In the final paper (Part III; Brázda & Palatinus, 2023*b* ▸), the problem of calibrating the distortions will be addressed and the application of the calibrations for obtaining accurate lattice parameters even for low-quality data will be shown.

## 2. Experimental

All theoretical developments presented in this work are illustrated on experimental 3D ED data. We used lutetium aluminium garnet (LuAG, Lu_3_Al_5_O_12_, 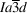 , *a* = 11.9084 Å at 100 K), which is very stable in the electron beam and provides high-quality data. A large monocrystal of LuAG was crushed in an agate mortar and the powder was suspended in water. A drop of the suspension was deposited on a Cu holey-carbon transmission electron microscopy (TEM) grid.

Data were collected on an FEI Tecnai G^2^ 20 transmission electron microscope operated at 200 kV with an LaB_6_ cathode, equipped with an Olympus SIS Veleta CCD camera (14 bit, 2048 × 2048 pixels) and a Nanomegas Digistar precession unit. The tilt step during the data acquisition was 1.0° and the α tilt ranges are given in Table S1 in the supporting information.

**Figure 1:**
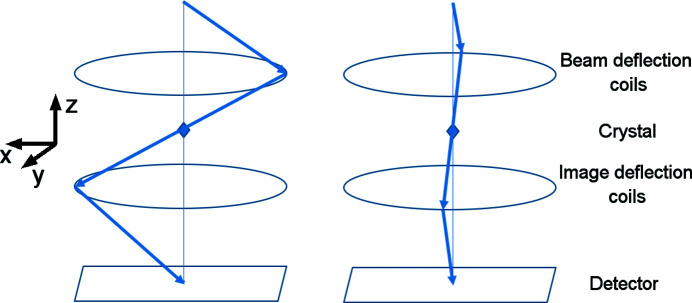
A schematic ray diagram of the two static electron beam experiments simulating a precession experiment (beam tilt and image tilt). The phases of the image tilt are 0° (tilt in the *xz* plane) and 90° (tilt in the *yz* plane).

If precession electron diffraction (PED) was used, the nominal precession angle was 1.0°. This value was more precisely determined during data processing. The PED method, which is often used in 3D ED experiments, is performed with a double conical beam-rocking system that uses double-deflection coils above (beam-deflection coils) and below (image-deflection coils) the specimen (Vincent & Midgley, 1994 ▸). In the process, the electron beam is precessed under a certain tilt angle with respect to the optical axis using the beam-deflection coils in the illumination lens system. Below the specimen, the displacement of the electron beam from the optical axis is compensated by using the image-deflection coils in the image-forming lens system (Fig. 1 ▸).

Data were measured with a Gatan cryo-tomography holder at 100 K to prevent contamination and to minimize possible beam damage. Data were recorded in microdiffraction mode with a 10 µm condenser lens aperture and at variable camera lengths. All data were processed with the software *PETS2* (Palatinus *et al.*, 2019 ▸).

More details about individual datasets used in this article are given in the supporting information, Section S1.

## 3. Optical distortions – theoretical aspects

A transmission electron microscope is an electron-optical system. All elements of the system, be it lenses, apertures, deflectors or stigmators, deviate from ideal optical elements and introduce aberrations to the image. While in TEM imaging the main concern caused by the presence of the aberrations is the decreased resolution, in diffraction the main concern is the shift in the position of the diffracted beams, *i.e.* geometric distortions of the diffraction pattern.

In the most general terms, each point *x*, *y* in the ideal diffraction pattern is shifted to *x*′, *y*′ in the experimental pattern due to the distortions, and our aim is to determine *x*′, *y*′ as a function of *x*, *y* with a small number of parameters describing the optical distortions.

**Figure 2:**
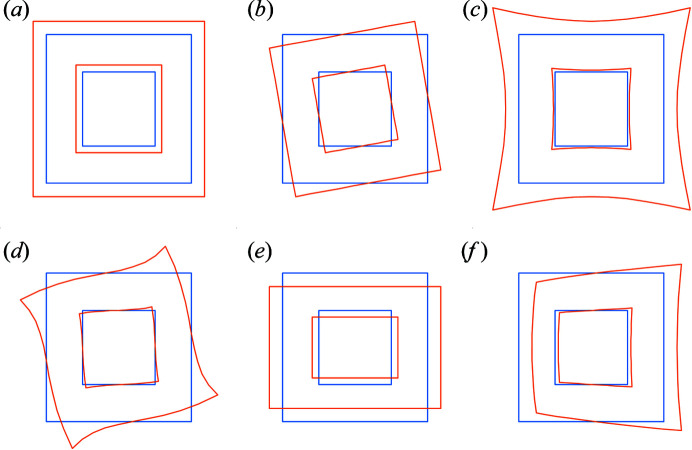
Distortions present in static beam electron diffraction data (blue denotes the undistorted pattern and orange the distorted pattern). (*a*) Magnification error, (*b*) in-plane rotation, (*c*) barrel-pincushion distortion, (*d*) spiral distortion, (*e*) elliptical distortion and (*f*) parabolic distortion.

The literature on optical distortions is quite rich, but it is mostly focused on distortions in the images (Williams & Carter, 2009 ▸; Rose, 2008 ▸; Hawkes, 2015 ▸; Krivanek *et al.*, 1999 ▸). Work focusing on distortions in the diffraction patterns commonly discusses only spiral, barrel-pincushion and elliptical distortions (Fig. 2 ▸). In order to have the most general description of the distortions, for which the common distortions are simple special cases, we use a general description of the distortions using trigonometric series. In this description, the distortion is decomposed into its radial and tangential component so that we have

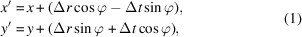

where Δ*r* is the radial component of the distortion and Δ*t* is the tangential component of the distortion (Fig. S1). φ is the azimuth of the point *x*, *y*: φ = arctan2(*y*, *x*). We also define *r* as the length of the vector (*x*, *y*): *r* = (*x* ^2^ + *y* ^2^)^1/2^.

Both components of the distortions are periodic functions of the azimuth, and they are thus conveniently expanded in a cosine series:

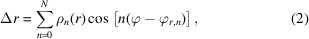




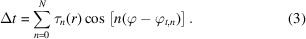

The coefficient *n* in the expansion represents the periodicity of the member of the series.

Finally, the functions ρ_*n*_(*r*) and τ_*n*_(*r*) express the dependency of the distortion on the length *r* of the vector (*x*, *y*). They can be conveniently expressed as polynomials:

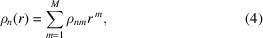




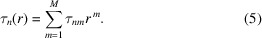

Such polynomial expansion (*m* is the degree of the polynomial) is commonplace in the analysis of optical aberrations, and it allows individual terms of the general expansion to be related to established ‘pure’ distortion types (see below).

Combined together, the general expressions of the radial and tangential distortions of a point (*r*, φ) become:

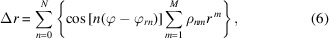




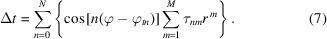

The parameters φ_*rn*_, φ_*tn*_, ρ_*nm*_ and τ_*nm*_ need to be determined either by calibration of the microscope or by refinement against diffraction data.

Note that *m* runs from 1, *i.e.* the polynomial does not have a constant term. This means that the point (0, 0) has no distortion. This point corresponds to the position of the optical axis of the microscope, where no geometric distortions are expected, and we denote it as the *centre of distortions*. The existence of a unique point with no distortion assumes that all the optical elements in the microscope are well aligned and share the same optical axis. In the following, we assume this premise holds.

**Figure 3:**
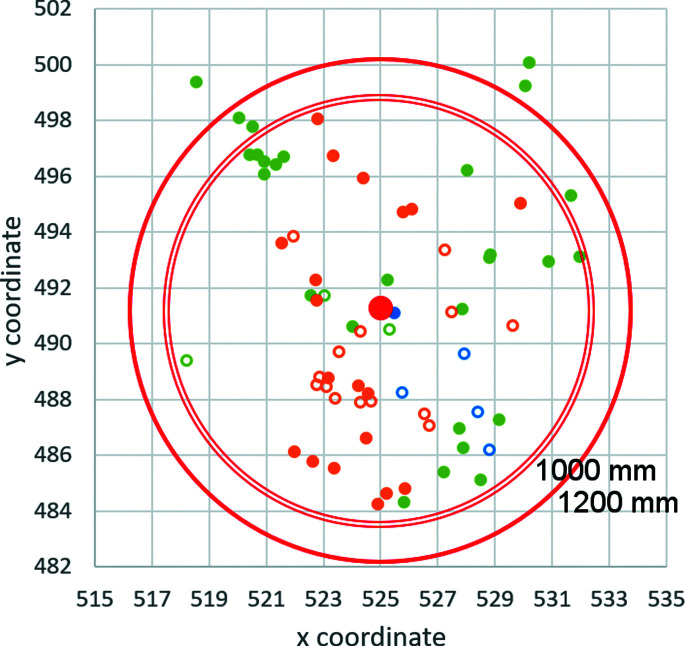
The centres of distortions refined against tens of 3D ED datasets for diffraction lens excitations equal to eucentric focus (green), 102.8% of the eucentric focus (orange) and 105.6% of the eucentric focus (blue) for camera lengths of 1000 mm (empty circles) and 1200 mm (full circles). The average position is marked by a red dot. The physical pixel size is 7.4 × 7.4 mm and the number of pixels is 1024 × 1024. The outer red rings mark distances of 0.03 Å^−1^ from the average position of the distortion centre for the two camera lengths.

The centre of distortions does not, in general, coincide with the position of the non-diffracted (primary) beam. The values *r* and φ in equations (1)–(7) [Disp-formula fd3] [Disp-formula fd4] [Disp-formula fd5] [Disp-formula fd6] [Disp-formula fd7] thus need to be calculated from the centre of distortions and not from the position of the primary beam. When the primary beam does not follow the optical axis, the distortions also affect its position. The position of the centre of distortions in the experimental data used in this work is stable and very close to the centre of the detector, although not exactly on the centre (Fig. 3 ▸). Its position is reproducible at different excitations of the lenses and varying beam and image tilts.

The coefficients of the distortions ρ_*nm*_ and τ_*nm*_ as defined in equations (4) and (5) have units of Å^−(*m*−1)^. However, as the numbers are small, it is more convenient – and in the case of elliptical distortion also customary – to provide the values in percent, without explicitly stating the units. We therefore adopt the convention of dropping the units and giving the values of the coefficients in percent. Formally, this can be introduced by defining normalized coefficients 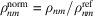 , with 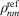 = 1 Å^−*m*−1^, and analogously for τ_*nm*_. 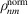 is then dimensionless and its value can be given in percent. In the following we drop the superscript ‘norm’ from the labelling of the coefficients.

The common image aberrations on one hand and diffraction pattern distortions on the other originate from the same physical effects and their mathematical descriptions are related. However, traditionally, the corresponding effects are named differently in imaging and in diffraction. These well known distortions correspond to specific values of *n* and *m* in equations (6) and (7), and possibly to a specific relationship between the coefficients, and they are summarized in Table 1 ▸. To the best of our knowledge, the distortion known in the field of imaging as coma (Smith, 2007 ▸) has not yet been described in the context of diffraction patterns and thus it does not have a commonly accepted name. In line with the tradition of naming the geometric distortions in diffraction by the typical shape they induce, we suggest naming this distortion *parabolic* distortion, because it changes a line perpendicular to the axis of the distortion to a parabola [Fig. 2 ▸(*f*)].

We have analysed the residual errors between the observed and expected positions of the reflections in our data after the refinement of the distortions listed in Table 1 ▸. Fig. S2 demonstrates the drop in the residual distances between expected and observed peak positions in dataset 1 (DS1) by more than an order of magnitude when the distortions are refined. Within the residuals, we have not been able to identify any other distortion with an amplitude higher than its estimated uncertainty. The next distortion, which is allowed for systems with a centre of symmetry, is *n* = 2, *m* = 3. This distortion should have the radial and tangential components equal in magnitude. The refinement in DS1 resulted in a radial amplitude equal to 0.008 (6)% and a tangential part equal to 0.003 (6)%, *i.e.* insignificant and much smaller than the amplitude of the other distortions. Interestingly, Hüe *et al.* (2005 ▸) seem to have identified a distortion with *n* = 2, *m* = 3 in their analysis of the transmission electron micrographs, thus in the imaging mode. Other distortions might be present in diffraction data from other microscopes. We also did not analyse the distortions potentially caused by image correctors or energy filters. The formulation given above [equations (6) and (7)] is, however, designed to be sufficiently general to allow for the description of these distortions too. In the following, we limit the discussion only to the six distortions presented in Table 1 ▸ and observed in our data. First, the well established distortions are briefly illustrated, then the parabolic distortion is introduced, and finally the effect of these distortions on PED data is revealed.

### 3.1. The ‘standard’ distortions

The first five distortions in Table 1 ▸, *i.e.* magnification, rotation, barrel-pincushion, spiral and elliptical, are well known. They can be related to the settings of the microscope (as will be discussed in detail in Part III of this article series), and they are always present to some extent in the data. If the lattice parameters are known, they can all be refined simultaneously against a single 3D ED dataset. See Section 4 for a discussion of a refinement with unknown lattice parameters.

As an illustration, Table 2 ▸ shows the parameters of these distortions refined against the datasets DS1, DS2 and DS3 (see Section 2 and Section S1 for a description of the datasets). These are three standard datasets collected during one session on three different crystals with careful alignment of the microscope, but without any specific attempt to minimize the distortions.

Without correction of these distortions, the lattice parameters differ from the expected cubic unit cell by up to 0.07 Å and 0.40° (see Section 5.1 for more details). This is because the 2D distortions deform the reconstructed reciprocal space (Fig. S3).

A few notes on the individual distortions follow.

(i) The magnification distortion correlates completely with the scaling of the lattice constant and cannot be determined without knowing the lattice parameters. This is due to the very short wavelength of electrons and thus a very flat Ewald sphere.

(ii) The rotation distortion is equivalent to a change in the orientation of the tilt axis. Refinement of the tilt axis is part of the data reduction step in most data processing programs, and this distortion is therefore in general not even considered as a distortion. However, as the orientation of the tilt axis is a result of the microscope setting and alignment, it is useful to consider it as a separate distortion that can be calibrated. Moreover, it correlates with the spiral distortion. Thus, without correcting for the spiral distortion, the refined position of the tilt axis will not be correct. Rotation distortion also correlates with the orientation of the unit cell in reciprocal space, and thus it correlates with the orientation matrix (Section 4.1), so simultaneous refinement of the rotation distortion and the orientation matrix may give biased results. Our experience is that the best results are obtained when the unit cell (orientation matrix) is refined with the spiral distortion while the rotation distortion is fixed. The correction for the spiral distortion then allows for the correct determination of the tilt axis. This workflow will be used in both worked examples presented with this article (detailed manuals for the examples are presented in the supporting information, Section S4). However, if we cannot or do not want to refine the orientation matrix, it is necessary to refine the rotation and spiral distortions together to arrive to the correct geometry.

(iii) The barrel-pincushion and spiral distortions are cubic distortions (*m* = 3). Thus, they are very small at low resolution, while increasing very steeply at higher resolution. As an example, the amplitudes ρ_03_ = 0.21 (1)% and τ_03_ = 0.47 (1)% obtained for DS1 (calibration constant 0.003693 Å^−1^ pixel^−1^) mean that a reflection with a resolution of 0.5 Å^−1^ would be shifted by 0.07 pixels radially and by 0.16 pixels tangentially, while a reflection with a resolution of 1.5 Å^−1^ would be shifted already by 1.92 pixels radially and 4.31 pixels tangentially.

(iv) The elliptical distortion is, in general, the main source of errors in the lattice parameters apart from mechanical instabilities. It is a linear distortion (*m* = 1). Even relatively small amplitudes of this distortion lead to appreciable changes in the lattice parameters (Section 4).

### 3.2. The parabolic distortion

The distortions described in the previous section are essentially always present in the data to some extent. This is not the case for the parabolic distortion. This distortion, being the diffraction counterpart of the coma, appears only when two conditions are met simultaneously:

(i) A shift of the beam away from the optical axis or the image tilt is applied to the ray path through the microscope column.

(ii) The diffraction lens is not exactly focused on the back focal plane.

The first condition is met typically when the crystal is tracked by the beam during a 3D ED experiment (Plana-Ruiz *et al.*, 2020 ▸), but it may also appear in techniques within the 4D scanning tunnelling electron microscopy (STEM) family (Ophus, 2019 ▸), serial ED (Bücker *et al.*, 2021 ▸; Smeets *et al.*, 2018 ▸; Wang *et al.*, 2019 ▸) or automated crystal orientation mapping (ACOM) (Rauch *et al.*, 2010 ▸; Rauch *et al.*, 2021 ▸). Image tilt is applied and hence this distortion is also induced in PED (Vincent & Midgley, 1994 ▸; Plana-Ruiz *et al.*, 2018 ▸). The second condition arises frequently in the micro- or nano­diffraction modes or in STEM mode, when a small probe size is used. The focus of the diffraction lens then needs to be adjusted to compensate for beam convergence and focus the diffraction pattern properly.

Both beam shift and image tilt lead to similar types of distortion. We discuss the beam-shift-induced distortions in the main text of this article, because it is present in all 3D ED geometries (static frames, continuous rotation, precession assisted). Beam-tilt-induced distortions give us a precious insight into the problems of precession-induced distortions. A thorough analysis of them may be found in the supporting information, Sections S2 and S3.

**Figure 4:**
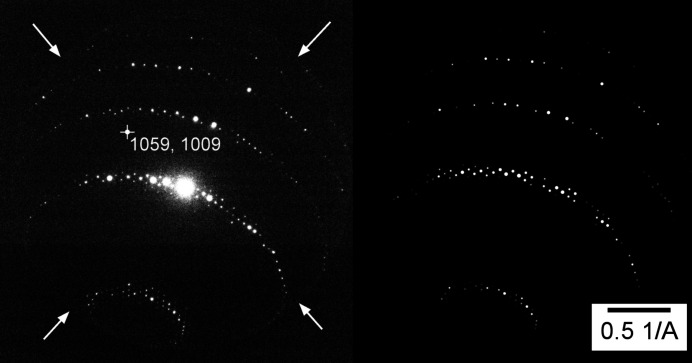
(Left) A diffraction frame and (right) its simulation, taken from dataset DS4. The arrows indicate an aperture limiting the field of view of the diffraction pattern, and the white cross with coordinates shows the refined position of the optical axis of the microscope (distortion centre) on the 2 k × 2 k detector.

For an illustration of the beam-shift-induced distortions we used DS4 collected to a very high resolution (>3 Å^−1^) and with a very high excitation of the diffraction lens (see Section 2 and Section S1 for details of DS4). Fig. 4 ▸ shows a diffraction pattern obtained in this experiment.

**Figure 5:**
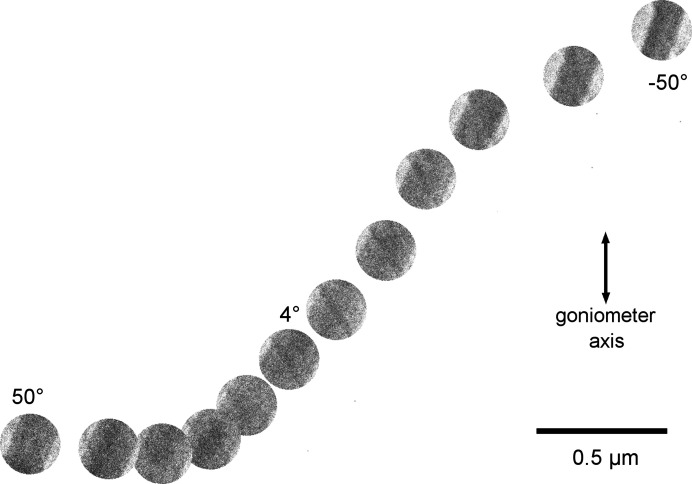
The movement of the crystal during data acquisition for DS4.

**Figure 6:**
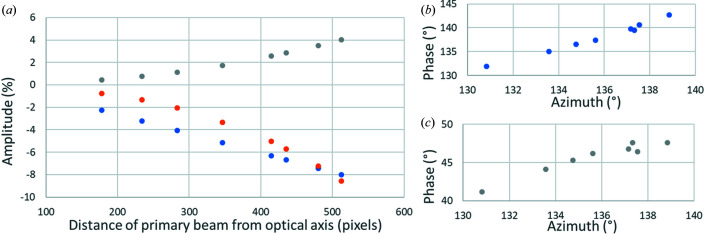
(*a*) The amplitudes of the magnification correction (orange), parabolic (blue) and elliptical (grey) distortions as a function of distance between the primary beam and the optical axis. (*b*) and (*c*) The dependence of the phases of (*b*) the parabolic (blue) and (*c*) the elliptical (grey) distortions on the azimuth of the difference vector of the primary beam from the optical axis.

During the collection of this dataset, the crystal was moving and the primary beam was shifted to follow the crystal (Fig. 5 ▸). As a result, the primary beam runs parallel to but away from the optical axis. We have divided the dataset into eight subsets, each containing 20 frames. The position of the primary beam within these subsets changed only to a limited extent and each subset can be approximately considered as coming from one shifted position of the primary beam. The refinement of distortions shows that, as the beam is shifted away from the optical axis, additional distortions appear as a function of the displacement of the primary beam from the optical axis. These distortions are (i) a change in magnification, (ii) parabolic distortion and (iii) additional elliptical distortion. The phases of the parabolic and elliptical distortions are very well aligned with the azimuth of the shift in the primary beam from the optical axis. Fig. 6 ▸(*a*) shows the evolution of the amplitudes of the magnification correction and parabolic and elliptical distortions as a function of the distance of the primary beam from the optical axis, and Figs. 6 ▸(*b*) and 6 ▸(*c*) show the dependence of the phases of the parabolic and elliptical distortions as a function of the azimuth of the shift vector.

### 3.3. Distortions in PED

**Figure 7:**
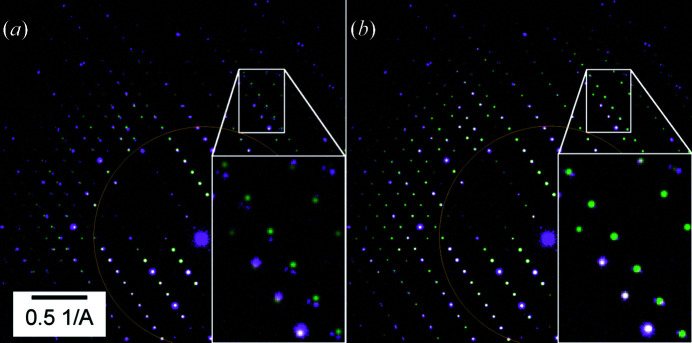
Comparisons of simulation (green) and experimental data (purple) for PED data (DS9) when parabolic distortion effects are (*a*) omitted and (*b*) considered. The overlaid brown circle outlines a resolution of 1 Å^−1^.

It was shown in Section 3.2 and Section S2 that the distortions (parabolic, elliptical and magnification correction) may change their amplitude and phase with the shift in the beam or tilt of the image. The changes are amplified by the excitation of the diffraction lens out of its eucentric focus. If PED mode is used, the beam and image tilt are constantly changing. As a result, the distortions are also changing. In particular, the phases of the elliptical and parabolic distortions change together with the phase of the precessing beam. It is shown in Appendix *A* [App appa] that, if the phase of the parabolic distortion is exactly equal to the phase of the image tilt, the image tilt dependent parabolic distortion leads to a specific distortion of the precession data, which shifts and splits the reflection position as a function of its excitation error according to the formulae

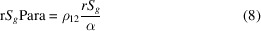




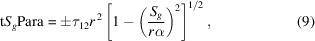

where *S*_*g*_ is the excitation error of the reflection, α is the precession angle expressed in radians and *r* is the distance of the reflection from the centre of distortions. r*S_g_* Para and t*S_g_* Para are the radial and tangential components, respectively, of the shift in reflection position induced by the parabolic distortion. The radial distortion thus changes linearly with excitation error, while the tangential term causes tangential splitting of the reflection. The splitting effect is clearly visible in the high-resolution part of the precession diffraction data (Fig. 7 ▸), and is often mistakenly attributed to wrong alignment of the precession itself. If the phase offset between the parabolic distortion and image tilt were 90°, the splitting would be caused by the radial part of the parabolic distortion (and thus it would be along the radial direction) and the tangential component would induce tangential reflection shift. Any other phase offset would cause both reflection shift and splitting originating from both radial and tangential parts of the parabolic distortion. Note that the amplitude of the tangential part of the parabolic distortion is three times smaller than the amplitude of the radial part (Table 1 ▸). Therefore, the effects caused by the radial part are three times more pronounced than those caused by the tangential part.

The image tilt dependent elliptical distortion in the data leads to a distortion given by the formulae (Appendix *A* [App appa])

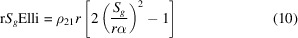




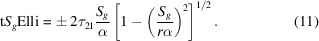

This term introduces a radial distortion, which is parabolic with excitation error, and reflection splitting that has opposite signs for the negative and positive excitation errors. In the case of elliptical distortion, a phase offset of 90° with respect to the image tilt phase would cause a change in the signs of the shift and splitting. A phase offset of 45° causes the splitting to occur in the radial direction and the shift in the tangential direction.

**Figure 8:**
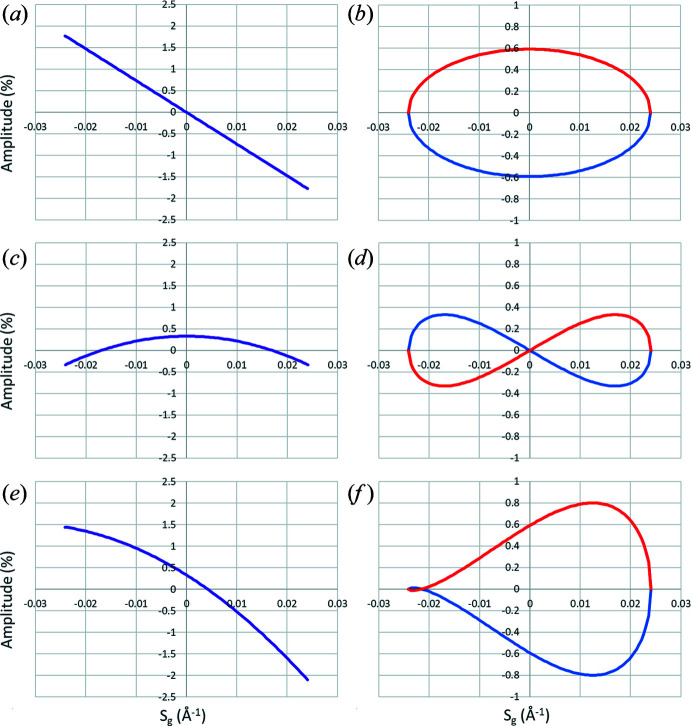
Excitation error (*S_g_*)-dependent effects of the image tilt induced parabolic and elliptical distortions on the position of a reflection, with the distance from the centre of the distortions equal to 1.5 Å^−1^ and with the amplitudes of the induced distortions the same as those found experimentally for the PED experiment DS8. For the sake of clarity, the phases of the distortions are set exactly to 0° so the radial components cause only reflection shifts and the tangential components cause only reflection splitting. The left-hand column shows the shift effects of the radial components of (*a*) the parabolic distortion, (*c*) the elliptical distortion and (*e*) their combination. Similarly, the right-hand column shows the splitting effects of the tangential parts of (*b*) the parabolic distortion, (*d*) the elliptical distortion and (*f*) their combination. Blue lines correspond to phases of the precessed electron beam between 0 and π, red lines correspond to phases between −π and 0, and purple lines correspond to phases between −π and π.

In the case of a zero phase offset for both parabolic and elliptical distortion, a combination of the terms arising from the parabolic and elliptical distortions results in an asymmetric splitting of the reflections, with smaller splitting for one sign of *S*_*g*_ (negative if both τ_12_ and ρ_21_ are positive) and larger splitting for the other sign of *S*_*g*_ (Fig. 8 ▸). As the average Δ*r* is not zero for a symmetric interval of *S_g_*, the elliptical distortion also introduces a net change in the average reflection position, which results in an additional magnification correction of −ρ_21_ *r*/3 (Appendix *A* [App appa]).

To illustrate these findings we analyse dataset DS8 collected with PED, precession angle 0.92°. The refined distortions are summarized in Table S2 (parts *A* and *C*). The experiment was done on the same crystal and with the same settings as dataset DS5 (no PED). Comparing these experiments, we can see that the magnification changed in the precession experiment, while the elliptical distortion remained the same. Because the observed beam shift induced parabolic distortion is small, the elliptical distortion corresponds almost entirely to the intrinsic one in both datasets.

In addition to these distortions, *S_g_*-dependent terms have appeared (Table S2 part *C*). These distortions have significant amplitudes, and an appreciably improved fit to the data can be obtained when the distortions are corrected (Fig. 7 ▸). This result shows the importance of distortion compensation and the general correctness of the applied model.

Further discussion of the relationship between the distortions induced in the beam tilt and image tilt (double tilt) experiment and in the precession-assisted data may be found in Section S3.

## 4. Distortions and lattice parameters

Optical distortions introduce deformations of the reconstructed 3D reciprocal space and thus they influence the obtained lattice parameters. Examples of these effects are demonstrated on a deformation of a cube in Fig. S3. It is possible to compensate for the effects of the distortions by calibration using a suitable material like LuAG. Thanks to this procedure it is possible to break the correlation between optical distortions and lattice parameters and obtain accurate lattice parameters even for materials with compromised diffraction data quality. The calibration procedures and the application of the obtained calibrations to the data will be described in Part III of this series. In this section we investigate the relationship and correlations between the distortions and lattice parameters, and show procedures which allow simultaneous refinement of the distortions and lattice parameters. We discuss three distinct cases: known lattice parameters and unknown distortion coefficients, unknown lattice parameters, unknown crystal system and unknown distortion coefficients, and finally unknown lattice parameters and unknown distortion coefficients, but known crystal system.

### 4.1. Case 1. Known lattice parameters

In this case the orientation matrix can be determined under the constraint of known lattice parameters. The coefficients of the distortions can then be determined to a good accuracy, as demonstrated in Section 5.1.

This is an ideal case, however, which is not always available. There are two main problems which do not allow lattice parameters to be obtained from other sources like powder X-ray diffraction. First, the material of interest may only be available in a very small quantity or it may only be a minor phase in the sample. Second, the lattice parameters may change during the experiment due to the accumulated electron dose, which is often observed for molecular crystals and other very beam-sensitive materials. These effects will be discussed in Part II of this series.

### 4.2. Case 2. Unknown lattice parameters, unknown crystal system and unknown distortions

This is the most challenging case. We need to determine simultaneously the orientation matrix and the coefficients of the distortions. This is a difficult task which is, in some cases, impossible to solve. Nevertheless, it is worth investigating it in detail. Two subcases are shown here, which differ in the number of crystals for which diffraction data are available.

#### 4.2.1. Only one crystal available

The distortions, if uncorrected, will result in the deformation of reciprocal space and, consequently, in distortion of the orientation matrix. An important question to answer is whether or not the deformation of reciprocal space due to the distortions is sufficiently nonlinear to be decoupled from refinement of the orientation matrix.

The standard approach to the determination of the orientation matrix is the least-squares refinement of its parameters that minimizes the distance between the predicted and experimental reflection positions. We minimize the function *S*,

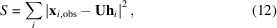

where the vectors **x**_*i*, obs_ are calculated from the reflection positions on the diffraction patterns and the positional angles of the crystal. **U** is the orientation matrix and **h**_*i*_ are the vectors of the reflection indices. If distortions are present, the correct vectors **x**_*i*, obs_ are not available, but distorted vectors **x**_*i*, dist_ are available instead. The refined matrix **U**_dist_ will be different from the correct matrix **U**. The difference can be expressed as **U**_dist_ = **LU**, where the matrix **L** describes the deformation due to distortions.

In practice, the above general expression needs to be modified slightly. Because the accuracy of the reflection position is much higher in the plane of the diffraction pattern than perpendicular to it, the distortions and also the unit cell can be most accurately determined if only the reflection positions in the plane of the diffraction pattern are considered. This means that before the vectors **x**_*i*, obs_ and **U** **h**_*i*_ are compared, they are projected onto the plane of the diffraction pattern. Appendix *B* [App appb] describes the derivation of the matrix **L** for the case of general distortion as well as for particular types of distortion.

The general expression for the deformation matrix **L** in the case of a single crystal is

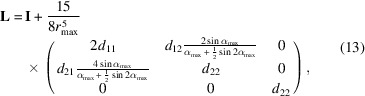

with *d*_*ij*_ being elements of the matrix **D** that contains the distortion coefficients,

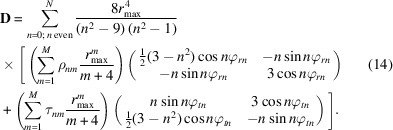

In the above expressions, *r*_max_ is the maximum resolution of the experimental data and α_max_ is the maximum tilt, *i.e.* the crystal is tilted between −α_max_ and α_max_ during the experiment. Although apparently complicated, the expression simplifies substantially for the typical distortions. As an example, for pure elliptical distortion with amplitude ρ_21_ and phase φ_*r*2_ we obtain

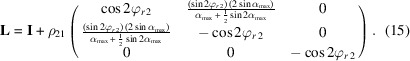

This matrix equals the unit matrix only in the (unrealistic) case of α_max_ = π and φ_*r*2_ = 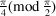 . In all other cases it introduces an appreciable error. An example with α_max_ = 60°, ρ_21_ = 0.2% and 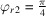 yields

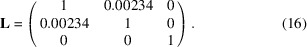

Despite the very moderate amplitude of the elliptical distortion, this matrix introduces an error of 0.27° between vectors (1 0 0) and (0 1 0). It also introduces a difference of 0.0071 in the length of Cartesian vectors (1 1 0) and (1 −1 0), *i.e.* a difference of 0.46%.

Matrices for other types of distortion are summarized in Appendix *B* [App appb], together with a discussion of their impact on the lattice parameters.

It is of the utmost practical importance to know whether the distortions are sufficiently nonlinear to allow a simultaneous determination of the orientation matrix and distortion co­efficients.

The distortions are, in general, a nonlinear function of the coordinates **x**_*i*_, while the deformation matrix **L** is linear. Thus, in principle, the distortion parameters and the orientation matrix **U** should be refinable simultaneously from the diffraction data. In practice, however, the distortions are correlated with the elements of **L**. It is desirable to have a means of quantification of this correlation, so that it can be estimated whether simultaneous refinement of the orientation matrix (and thus unit-cell parameters) and distortion coefficients is possible and reliable, or if the correlation prevents a reliable combined refinement. The correlation can be expressed by means of the standard Pearson correlation coefficient ρ(**L**, *d*). This coefficient is derived in Appendix *B* [App appb] for the general form of matrix **L**.

The second important quantity is the residual error in reflection positions that cannot be explained by the matrix **L**. If this error is very small, then the refinement will not be sensitive to the simultaneous refinement of the distortions and the orientation matrix. This error can be expressed as the root-mean-square deviation (RMSD) of the reflection positions for a given distortion. RMSDs for various types of distortion are also derived in Appendix *B* [App appb].

Individual distortions lead to the correlation coefficients and RMSDs shown in Tables 3 ▸ and 4 ▸, with least-squares-optimized matrix **L**.

**Figure 9:**
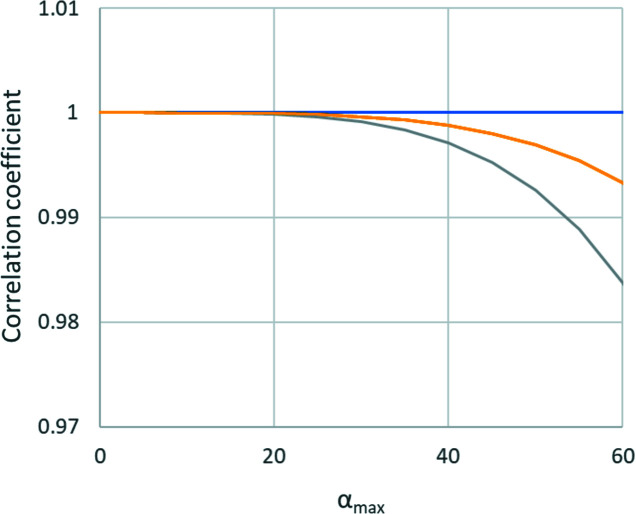
Correlation coefficients between the elliptical distortion and refinement of the orientation matrix, plotted for elliptical distortion phases 0° (blue), 20° and 70° (yellow), and 45° (grey).

Although the differences between the correlation coefficients may seem small and all of them appear high, in practice a distortion with a correlation coefficient as high as 99% can be refined against good 3D ED data, as long as the residual RMSD is a sufficiently high fraction of the RMSD induced by experimental noise. Thus, Tables 3 ▸ and 4 ▸ indicate that most distortions can be refined. However, the magnification distortion is perfectly correlated; it cannot be refined and magnification must be calibrated. A particular case is the elliptical distortion. Fig. 9 ▸ shows a plot of the dependence of the correlation coefficient on the maximum tilt angle α_max_ and the phase of the elliptical distortion. The plot illustrates that ρ(**L**, *d*) is very high in all cases, and for φ_*r*2_ = 0 it remains 100% regardless of α_max_. Because the phase of the elliptical distortion is not fixed, a component with φ_*r*2_ = 0 always correlates with the refinement of the orientation matrix, and elliptical distortion can never be reliably refined together with the unconstrained refinement of the orientation matrix.

#### 4.2.2. Multiple crystals available

If multiple crystals of the same phase (*i.e.* with identical lattice parameters) are measured at different orientations of the crystal with respect to the microscope, then the data can be combined and the effect of distortions can be, to a large extent, decoupled from the refinement of the orientation matrix. Expressed quantitatively, assume that the orientation of crystal *n* is related to the reference orientation by a rotation matrix **R**_*n*_ such that the orientation matrices are related by **U**_*n*_ = **R**_*n*_ **U**_1_. Assuming the microscope distortions are equal for both experiments, the deformation matrices **L**_1_ and **L**_*n*_ will be related by 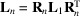 . A deformation matrix resulting from a combined refinement against all crystals can be obtained in a way analogous to the case of only one crystal [Appendix *B* [App appb], equation (46)]. The correlation coefficient can also be evaluated for such a combined deformation matrix.

Intuitively, if the orientations of the crystals involved in the refinement are sufficiently different, the correlation between the (common) lattice parameters and the distortions will be substantially decreased. We illustrate this quantitatively for the case of two crystals mutually rotated by 90° around **z**. The matrix **L** for this case is derived in Appendix *B* [App appb], equation (64).

**Figure 10:**
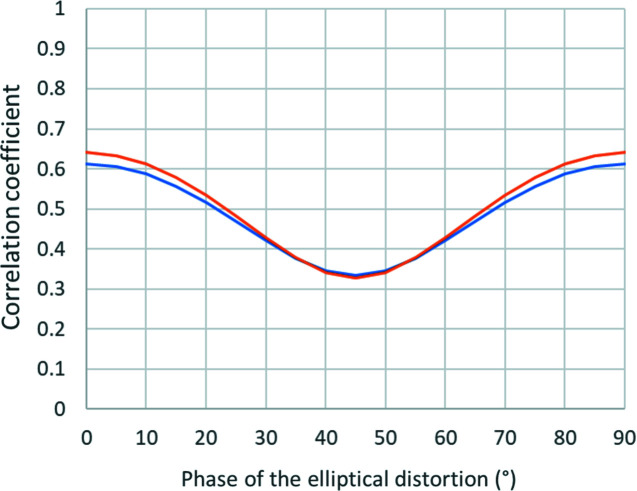
The correlation coefficient for the elliptical distortion as a function of the phase of the elliptical distortion in the case of two crystals mutually rotated by 90° for α_max_ of 20° (blue) and 60° (orange).

Fig. 10 ▸ shows a plot of the correlation coefficient as a function of α_max_ and the phase of the elliptical distortion. In this particular case, the correlation coefficient never exceeds 0.65 and is essentially independent of the tilt range. The refinement of lattice parameters and distortions is thus easily possible and robust. Section 5.1 shows an example of such a combined refinement.

### 4.3. Case 3. Known or reasonably assumed crystal system

Here **U** can be determined under the constraints of the known crystal system. The strength of such constraints depends, obviously, on the crystal system, and also on the orientation of the investigated crystal and the nature of the distortions.

**Figure 11:**
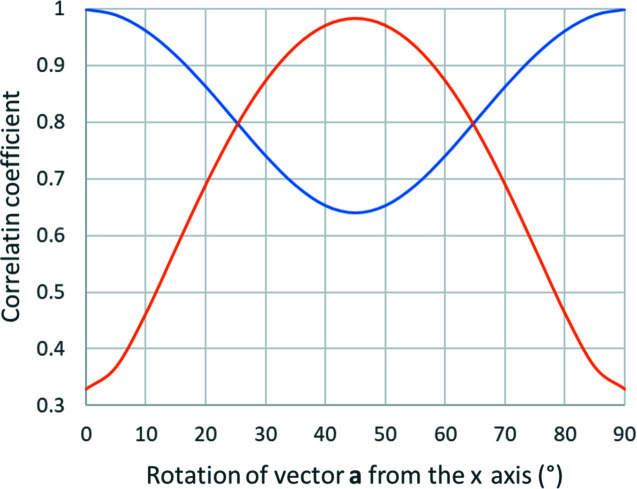
The correlation coefficient for the elliptical distortion for refinement under constraint of an orthorhombic crystal system. The horizonal axis represents the rotation of the crystal lattice vectors away from the orientation **a** || **x**. The phases of elliptical distortion 0° (blue) and 45° (orange) are shown and α_max_ was 60°.

Mathematically, this case is similar to the previous case, but with the function *S* minimized under the constraint that the lattice parameters must obey the restrictions given by the crystal system. This can be achieved by applying the method of undetermined Lagrange multipliers as shown in Appendix *B* [App appb]. A simple illustrative example is the case of an orthorhombic crystal system with the lattice vector **c** parallel to **z**, and vectors **a** and **b** rotated in the *xy* plane by an angle θ. Although the deformation matrix can be determined analytically, the expressions are very complicated. Here we therefore give only the plots of the resulting correlation coefficient for the phase of elliptical distortion 0° and 45° and α_max_ of 60°, and for various orientations of the crystal axes with respect to the reference coordinate system (Fig. 11 ▸). The plot shows that if the crystal axes are perfectly aligned with the reference coordinates (θ = 0°) and the phase of the elliptical distortion is 0°, then the correlation coefficient is still 100%. However, as soon as the crystal is rotated by only a few degrees, the correlation drops. Thus, under symmetry constraints, the vast majority of crystal orientations allow the refinement of elliptical distortion, and only extremely special circumstances lead to perfect correlation.

Some examples of this case are shown and discussed in Section 5.1.

## 5. Case studies

### 5.1. Example 1. Breaking of the correlation between elliptical distortion and lattice parameters

*Accurate lattice parameters using single- and multi-crystal approaches*.

A detailed description of the refinements may be found in Example 1 in the supplementary information. Datasets DS1, DS2 and DS3 from three different crystals were measured under the same conditions. Lattice parameters for the distorted unit cells (no distortion corrections were applied during the refinement) are summarized in Table 5 ▸.

Using single datasets for the determination of accurate lattice parameters does not lead to convergence because of the almost perfect correlation between lattice parameters and elliptical distortion. We need either to use known lattice parameters from X-ray powder diffraction (XRPD) (Section 5.1.1), assume a Bravais lattice (Section 5.1.2), or combine these crystals into one dataset and refine the elliptical distortion (Section 5.1.3) to determine the correct lattice parameters.

#### 5.1.1. Known lattice parameters

Application of the known cell from XRPD, which was fixed during the distortion refinement, yielded the distortions for the three crystals given in Table 2 ▸. The distortions were then fixed and the unit cell was refined without any restrictions (Table 6 ▸) to show that the deviations of the lattice constant *a* from 11.9084 Å and of the angles from 90° as shown in Table 5 ▸ are due to optical distortions.

#### 5.1.2. Assumed Bravais lattice

We have assumed a monoclinic Bravais lattice to show the power of the unit-cell symmetry constraint (monoclinic angle β). The unit-cell setting was chosen on purpose in each dataset so that the angle with the largest deviation from 90° was selected as the monoclinic angle. This is the worst-case scenario – a poorly fitting angle is not constrained by the monoclinic symmetry. The last step in the unit-cell refinement was fixing the obtained distortion corrections and refining the unit cell without any constraint. Monoclinic constraint on the unit-cell symmetry (Table 7 ▸) produced only slightly worse lattice parameters than the very strong constraint using a known unit cell (Table 6 ▸). When we compare the elliptical distortion parameters (Table 8 ▸) obtained from the monoclinic cell constraint and known cell constraint we can see that for DS1 and DS2 the values of the amplitude differ by less than 10%, while for DS3 it differs by about 20%. Thus, using a much less strong monoclinic symmetry constraint is enough to bring the value of the elliptical distortion very close to its correct value. However, note that, for some crystal orientations, even quite high symmetries (all except cubic) do not warrant a successful refinement of the elliptical distortion (See Section 4.2)

#### 5.1.3. Combination of crystals

*Combination of the three crystals into one dataset*.

The combination proceeded as follows. Each dataset was processed separately, and its orientation matrices were refined without any distortion correction. The datasets were then merged into one using a procedure in *PETS2*, ‘Merge projects’. This procedure uses the known orientation matrices and the positional angles of frames to transform all but the first dataset (in this case DS2 and DS3) so that they correspond to the orientation matrix of the first data set (DS1 in this case). After the merging, all frames could be processed jointly. The simultaneous refinement of the lattice parameters and distortions against this merged dataset without any constraints resulted in *a* = 11.904 (1) Å, *b* = 11.902 (1) Å, *c* = 11.907 (1) Å, α = 89.92 (1)°, β = 90.07 (1)° and γ = 89.89 (1)°. The elliptical distortion was equal to 0.351 (4)% and 65.9 (3)°, barrel-pincushion distortion equalled 0.202 (6)% and spiral distortion refined to 0.462 (4)%. This result is much better than the free refinement against a single dataset, but it is not perfect. The reason is that the merging of the datasets is based on knowledge of the orientation matrices, which in turn depends on the distortions. Without distortion corrections, the orientation matrices are inaccurate and the merging of the datasets is affected by this inaccuracy. The significant deviations of the lattice angles from 90° are caused by this inaccuracy. The result can be substantially improved by using the obtained distortion parameters as calibration values for the reprocessing of individual datasets. This leads to improved orientation matrices and an improved merging process. This iterative approach results in the lattice parameters *a* = 11.906 (1) Å, *b* = 11.910 (1) Å, *c* = 11.907 (1) Å, α = 90.03 (1)°, β = 90.01 (1)° and γ = 89.99 (1)°, *i.e.* essentially perfect cubic parameters. The elliptical distortion refined to 0.388 (3)% and 67.3 (2)°, barrel-pincushion distortion to 0.204 (4)% and spiral distortion to 0.409 (3)%.

Another, simpler, possibility to improve the accuracy of the lattice parameters without the need for iterative refinement of the optical distortions and the orientation matrix is to improve the orientation angles of the particular diffraction frames directly in the merged dataset. This can be done using the frame orientation procedure, which compensates for the imperfections introduced in the frame orientations by the distorted orientation matrices. This option will be extensively discussed and demonstrated in Part II of this article series.

### 5.2. Example 2. Distortions in precession data

*Effects of distortions in precession data with a diffraction lens excited from its eucentric focus*.

This example uses dataset DS9. The measured crystal was placed at the eucentric height of the stage, on the goniometer tilt axis, and it was focused with the objective lens. Because the beam was slightly convergent, the spots were broadened into very small discs. The diffraction lens (DL) was excited to 105.7% of its eucentric focus to focus the diffraction pattern and turn the discs into sharp spots. The precession unit was carefully aligned. A detailed description of the dataset refinement may be found in Example 2 in the supplementary information.

**Figure 12:**
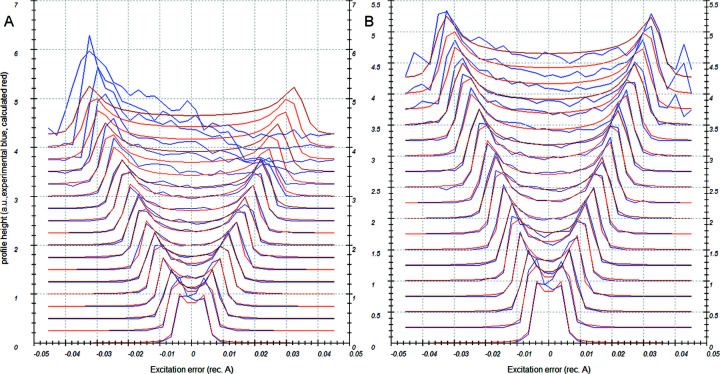
Rocking curves (blue – experimental, red – calculated) of DS9 data treated (*a*) without compensation of the parabolic distortion effects and (*b*) with compensation.

Without the correction for the *S*_*g*_-dependent distortions, the refinement of the distortions under the cubic symmetry constraint results in a barrel-pincushion distortion of −0.201 (5)% instead of the expected −0.444 (6)%. Elliptical and spiral distortions converge close to the expected values. The incorrect value of the barrel-pincushion distortion, together with the image demagnification caused by the parabolic distortion induced by the precession (see Section 3.3), result in an incorrect lattice parameter *a* = 12.001 Å instead of the correct 11.908 Å. The predicted positions of the diffraction maxima do not match well with the experimental data when the *S_g_*-dependent distortions are omitted, especially at larger resolution [Fig. 7 ▸(*a*)]. After the refinement of the radial *S_g_* Para coefficient, which describes the decisive majority of the diffraction position shifts due to the parabolic distortion, the match becomes much better [Fig. 7 ▸(*b*)]. Fig. 12 ▸ shows that without the compensation of the effects of the parabolic distortion it is not possible to integrate the diffraction data properly. The radial *S_g_* Para coefficient converged to −1.016 (3)% and the barrel-pincushion coefficient converged to −0.418 (5)%. Based on our experience, the amplitude of the magnification correction of the precession data in comparison to the data without precession is approximately equal to one half of the radial *S_g_* Para coefficient. For this dataset the magnification correction is equal to −0.587 (6)% (thus 0.58 times the r*S_g_* Para) as determined by the refinement with lattice constants obtained from X-ray powder diffraction.

## 6. Conclusions

Accurate determination of the orientation matrix from 3D ED data is crucial for obtaining accurate lattice parameters, as well as for accurate integration of the intensity data. In this work we have analysed thoroughly the effect of optical distortions induced by the optical elements of the transmission electron microscope on the reflection positions and thus also on the accuracy of the lattice parameters. A new type of distortion, the parabolic distortion, is described, and it is shown to be important under some circumstances. The parabolic distortion induces excitation-error dependent reflection shift and splitting when electron diffraction data are collected with PED.

The shifts in reflection positions caused by optical distortions lead to inaccurate lattice parameters. A detailed analysis of the relationship between the optical distortions and the distortion of the orientation matrix shows that all distortions except for magnification and elliptical can be easily determined from a single 3D ED dataset, together with the parameters of the orientation matrix. However, the magnification distortion correlates perfectly with scaling of the lattice parameters, and the magnification thus always needs to be carefully calibrated. Similarly, the component of the elliptical distortion parallel to the rotation axis correlates perfectly with the deformation of the orientation matrix when both are simultaneously refined without any constraints, and the elliptical distortion thus cannot be refined freely together with unrestrained refinement of the orientation matrix. However, if knowledge of the crystal system is used, or if more than one crystal is used for the refinement, the elliptical distortion can also be determined and corrected for and, consequently, the lattice parameters can be determined to a good accuracy.

Optical distortions are not the only possible reason for inaccurate values of the lattice parameters. In the second part of this miniseries, we will analyse other sources, especially the mechanical instabilities of the instrument and the effects of radiation damage. Optical distortions may also be calibrated to a good accuracy. The calibration then allows an accurate determination of lattice parameters even from data which, due to their limited quality, may not permit a full independent determination of all distortion coefficients. The calibration of all distortions discussed in this paper will be described in the last part of the miniseries.

## Supplementary Material

Additional figures and tables, plus manuals for Examples 1 and 2. DOI: 10.1107/S2052252522007904/zu5001sup1.pdf

Raw data and PETS2 input files for examples 1 and 2.: https://doi.org/10.5281/zenodo.6424241

## Figures and Tables

**Table 1:** Relationship between standard distortions and the coefficients of the general description

Name of the distortion in the diffraction pattern	Name of the equivalent aberration in the image	Radial component	Tangential component
Magnification correction	–	*n* = 0, *m* = 1	–
In-plane rotation	–	–	*n* = 0, *m* = 1
Barrel-pincushion	Spherical aberration	*n* = 0, *m* = 3	–
Spiral	–	–	*n* = 0, *m* = 3
Elliptical	Second-order astigmatism	*n* = 2, *m* = 1	*n* = 2, *m* = 1, τ_21_ = ρ_21_, φ_*t*2_ = φ_*r*2_ − π/4
Parabolic	Coma	*n* = 1, *m* = 2	*n* = 1, *m* = 2, τ_12_ = ρ_12_/3, φ_*t*1_ = φ_*r*1_ − π/2

**Table 2:** Refined distortion parameters for datasets DS1, DS2 and DS3

	DS1		DS2		DS3	
Distortion	Amplitude (%)	Phase (°)	Amplitude (%)	Phase (°)	Amplitude (%)	Phase (°)
Magnification	−0.016 (9)	–	0.031 (10)	–	0.020 (11)	–
Barrel-pincushion	0.211 (7)	–	0.201 (8)	–	0.200 (8)	–
Spiral	0.463 (7)	–	0.446 (7)	–	0.452 (8)	–
Elliptical	0.394 (4)	67.7 (3)	0.391 (4)	67.0 (3)	0.382 (4)	67.3 (3)

**Table 3:** Correlation coefficient ρ(**L**, *d*) for individual standard distortions

Distortion	General expression for ρ(**L**, *d*)	Value for α_max_ = 60°
Magnification	1	1
Rotation	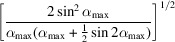	0.984
Barrel-pincushion	0.958	0.958
Spiral	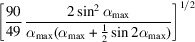	0.943
Elliptical	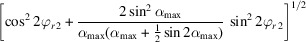	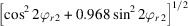

**Table 4:** Root-mean-square deviations RMSD(**L**, *d*) for individual standard distortions

Distortion	General expression for RMSD(**L**, *d*)	Value for α_max_ = 60°
Magnification	0	0
Rotation	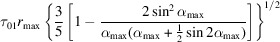	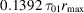
Barrel-pincushion	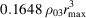	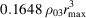
Spiral	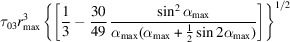	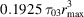
Elliptical	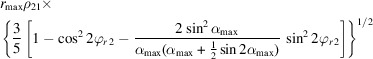	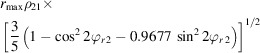

**Table 5:** Lattice parameters from unconstrained refinement without distortion compensation

	*a* (Å)	*b* (Å)	*c* (Å)	α (°)	β (°)	γ (°)
DS1	11.848 (1)	11.858 (3)	11.907 (1)	90.21 (1)	90.33 (1)	89.97 (1)
DS2	11.842 (1)	11.881 (2)	11.888 (2)	89.77 (2)	89.75 (1)	89.75 (1)
DS3	11.854 (1)	11.863 (3)	11.890 (1)	89.80 (1)	90.40 (1)	90.11 (1)

**Table 6:** Lattice parameters from unconstrained refinement with distortion compensation obtained from known unit-cell constraint

	*a* (Å)	*b* (Å)	*c* (Å)	α (°)	β (°)	γ (°)
DS1	11.909 (1)	11.910 (2)	11.908 (1)	90.00 (1)	90.00 (1)	90.02 (1)
DS2	11.908 (1)	11.914 (2)	11.908 (2)	90.03 (2)	90.00 (1)	89.98 (1)
DS3	11.907 (1)	11.912 (2)	11.909 (1)	90.001 (3)	90.00 (1)	90.00 (1)

**Table 7:** Free unit-cell refinement with distortions obtained from monoclinic unit-cell symmetry constraint induced during simultaneous unit-cell and distortion refinement

	*a* (Å)	*b* (Å)	*c* (Å)	α (°)	β (°)	γ (°)
DS1	11.917 (1)	11.913 (3)	11.903 (1)	90.00 (1)	90.02 (1)	90.00 (1)
DS2	11.899 (1)	11.915 (2)	11.909 (2)	90.02 (2)	90.03 (1)	89.99 (1)
DS3	11.900 (1)	11.910 (3)	11.909 (1)	90.00 (1)	90.08 (1)	90.01 (1)

**Table 8:** Comparison of elliptical distortion parameters obtained from two different constraints: monoclinic symmetry of the unit cell and known unit-cell parameters from X-ray powder diffraction

	Elliptical distortion from monoclinic cell constraint		Elliptical distortion from known cell constraint	
	Amplitude (%)	Phase (°)	Amplitude (%)	Phase (°)
DS1	0.416 (14)	64.8 (10)	0.395 (4)	67.7 (3)
DS2	0.384 (10)	70.5 (18)	0.391 (4)	67.1 (3)
DS3	0.313 (18)	67.5 (20)	0.383 (4)	67.3 (3)

## Appendix A Effect of distortions on precession diffraction data

Our input assumptions are that the image tilt induces distortions with phase dependent on the direction and amplitude of the deflection.

For simplicity, let us consider only the most prominent distortions, parabolic (radial and tangential components ρ_12_, τ_12_), elliptical (radial and tangential components ρ_21_, τ_21_) and their corresponding phases. Let us define the deflection (tilt) in the *x* direction as a tilt with zero phase. Then the radial and tangential first-order distortions are defined as

Similarly, the second-order distortions are given by

As the beam precesses, the direction of the tilt precesses around the central axis, and its momentary direction is defined by the precession phase θ. The distortions of a reflection with distance from the optical axis *r* and azimuth φ are, for a given θ, defined by

The change in the excitation error of a reflection with the precession is

where α is the precession angle. Assuming very sharp reflections, the reflection with excitation error *S*_*g*_ is in the diffraction position only if

*i.e.* for

Inserting this expression for θ into the expressions for the distortions, we get the final general expressions,

The ± term in front of the arccos causes splitting of the reflections. The average position is obtained as the average of the two branches. We then get the average distortions in this form:

These two expressions are dominant in the observed data.

For the elliptical distortion, we obtain:

Note that the amplitude and phase terms can be combined into an effective single parameter. That means that the distortions in precession do not allow (and also do not need) the determination of the amplitude and phase term separately. However, if the radial and tangential parts of the distortion have a known relationship, as is the case for the parabolic and elliptical distortions, the amplitude and phase of the distortion can be calculated from the refined *r* and *t* coefficients.

If the phases of the distortions have special values, these general expressions simplify further, namely if

For the tangential phases equal to 90° and ±45° the respective average tangential distortions vanish.

The r*S_g_* Para and t*S_g_* Para terms are symmetrical about *S_g_* = 0, and thus they do not induce any overall change in magnification or rotation when averaged over *S_g_*. This is not the case for the r*S_g_* Elli and t*S_g_* Elli terms. The average radial shift of a reflection subject to the distortion by r*S_g_* Elli is given by

Thus, the non-zero r*S_g_* Elli coefficient also induces an additional magnification distortion with amplitude  . Analogously, a non-zero t*S_g_* Elli coefficient would induce an additional rotation distortion with amplitude  .

## Appendix B Derivation of relationships for the correlation of distortions and cell deformation

### b1. Introduction

Any optical distortion shifts the positions of reflections on the diffraction pattern and thus also shifts the recalculated coordinates in 3D reciprocal space. If a lattice is least-squares-fitted into such a deformed set of coordinates, deformation of the lattice or its orientation may follow. In this appendix we will investigate the effects of distortion on the deformation of the fitted lattice.

### b2. Notation

In the following, vectors are considered as 3×1 matrices. Transposed vectors (*e.g.* **v** ^T^) are considered a row vector, *i.e.* a 1×3 matrix.

**u** : A general vector in the diffraction pattern plane. **u** is considered a vector with three components, *x* and *y* corresponding to the coordinates on the diffraction image and *z* equal to 0. This corresponds to ignoring the curvature of the Ewald sphere in the calculation. This approximation simplifies the calculations and it does not introduce any significant error to the result.

**R**_*c*_ : A rotation matrix rotating the crystal *c* to such a position that the orientation of its reciprocal lattice coincides with the reference reciprocal lattice. If only one crystal is analysed **R**_*c*_ is irrelevant and can be set to the identity matrix, but it needs to be considered if the effect of combining more crystals is investigated.

**R**_α_ : A rotation matrix by an angle α around the *x* axis. Brings the coordinates on the diffraction plane to the coordinates in the reference Cartesian coordinate system.

**u**_i_ : An ideal positional vector of a reflection in the diffraction plane, with no deformation.

**u**_d_ : A distorted vector in the diffraction plane. **u**_d_ = **u**_i_ + Δ**u**, or, using the definitions from the main text [equations (1)–(5) [Disp-formula fd3] [Disp-formula fd4] [Disp-formula fd5]], **u**_d_ = (*x*′, *y*′, 0).

**x**_i_ : An ideal positional vector of a reflection in the 3D Cartesian coordinate system.  .

**x**_d_ : A distorted vector in 3D reciprocal space.  .

**U**_i_ : The ideal orientation matrix. Would be obtained by least-squares fitting to the set of **x**_i_.

**U**_d_ : The distorted orientation matrix. Would be obtained by least-squares fitting to the set of **x**_d_.

**L** : The deformation matrix due to the distortion. Defined as **U**_d_ = **L** **U**_i_. Finding the matrix **L** for various types of distortion is the main purpose of this appendix.

Δ**L** : The deviation of **L** from the unit matrix; Δ**L** = **L** − **I**.

**x**_*L*_ : A reflection position in 3D reciprocal space after application of **L**.  . This is the approximation of **x**_d_ obtained by the linear deformation of **x**_i_ by **L**.

**u**_*L*_ : A positional vector of a reflection in the diffraction plane after the application of matrix **L**. **u**_*L*_ =  . **P** is a projection matrix that projects the vector onto the *xy* plane. **u**_*L*_ is the approximation of **u**_d_ after the linear deformation by **L**.

### b3. Derivation of matrix **L** – case without symmetry constraints

A naïve approach to finding the matrix **L** could be minimizing the total sum of squared differences between **x**_*L*_ and **x**_d_,  , where the sum runs over all measured reflections. This approach, however, requires knowledge of the 3D position of each reflection. While the position on the diffraction pattern plane is known to a very good accuracy, the position perpendicular to the plane is known to a much lower accuracy, due to many effects ranging from crystal imperfections through the uncertainties in crystal orientation to the use of PED. More accurate results can thus be obtained if only the positions in the diffraction plane are used in the minimization. Thus, we minimize the function

Inserting the definitions of the vectors **u**_*L*_ and **u**_d_ we get

Function *S* is a function of the elements of **L** and can be minimized by setting its derivative over all elements equal to zero:

Using the notation Δ**L** = **L** − **I** and **u**_d_ = **u**_i_ + Δ**u**, this can be rewritten as

We denote the first part in brackets, on the left-hand side of this equation, as **A** and the second part in brackets as **B**.

The above matrix equation constitutes a set of nine linear equations for the nine elements of Δ**L**, from which Δ**L** can be solved and subsequently **L** can be obtained as **L** = **I** + Δ**L**.

If only one crystal is considered, **R**_*c*_ can be set to the unit matrix and removed from the equation. If, however, more than one crystal is included in the calculation, each with its own matrix **R**_*c*_, then the minimized function runs over all reflections of all crystals:

The resulting equation is equivalent to equation (44), just with both sides summed over the involved crystals, *i.e.*

In the following we will find explicit expressions for **A** and **B**. Their exact values depend on the lattice parameters and crystal orientation. However, we may find useful crystal-independent approximations by considering the limit of an infinitely large unit cell, which is equivalent to replacing the sums in **A** and **B** by integrals. The expressions for **A** and **B** then become

and

The integration runs over a circle in the diffraction pattern plane up to the maximum resolution *r*_max_ and over the angular range covered by the experiment. For simplicity, we will assume that the angular range is symmetric around 0 and runs from −α_max_ to α_max_. Generalization to nonsymmetric ranges is straightforward through the application of a suitable rotation matrix **R**_*c*_.

It is convenient to express the integral in spherical coordinates. The integral **A** can then be explicitly written as

Here, for simplicity of notation, we have introduced  .

To simplify the representation of the result, we further define

We can then write

Here, *l*_*i* *j*_ are the elements of Δ**L**_*c*_. We can see that elements *l*_23_ and *l*_32_ occur only as a sum and they thus cannot be determined independently. This is an expected result. As we are projecting onto the diffraction plane, a small rotation around the rotation axis does not change **u**_*L*_. We thus have to fix this rotation.

When evaluating the integral **B**, we use the relationship

Inserting this explicit form into the expression for **B** and expressing the integral in spherical coordinates by analogy with **A** results in

The integrals in square brackets can be evaluated if expressions for Δ*r* and Δ*t* [equations (6) and (7)] are used. Because the third row and third column of the inner matrix are zero, we will consider only the 2×2 matrix of non-zero coefficients. We will denote this matrix **D**. The evaluation of **D** is lengthy but not complicated. The result is

Finally, using elements of matrix **D**, the integral **B** can be expressed as

For one crystal we can solve the equation **A** = **B** for the elements of **Δ** **L**_*c*_. We obtain

If more than one crystal is used, equation (46) needs to be used to construct the equations and solve them for Δ**L**.

For only one crystal, the value of *l*_23_ is not determined by the equations, which corresponds to the non-determined rotation around the tilt axis. With only one crystal and with **R**_*c*_ = **I**, the value of *l*_23_ can be set arbitrarily and we set it equal to zero. With more than one crystal with different mutual orientations, the ambiguity can be solved.

If the distortion coefficients are known, matrix **L** can be used to ‘undo’ the effect of distortions from the orientation matrix refined against distorted data.

In the following, we will consider the case of only one crystal. It is very instructive to investigate the form of the matrix **L** for specific distortions. Here we provide some of them. Some results are trivial, while others provide useful insights.

(i) Parabolic distortion (*n* = 1, *m* = 2).

Matrix **D** is zero for odd *n*. Thus, the odd-order distortions, including the parabolic distortion, do not induce deformation of the unit cell.

(ii) Scaling error (radial only, *n* = 0, *m* = 1),

As expected, a radial linear distortion scales the unit cell by 1 + ρ_01_.

(iii) Barrel-pincushion distortion (radial only, *n* = 0, *m* = 3),

This distortion increases the diagonal elements of **L** if ρ_03_ > 0, effectively scaling down the direct-space lattice parameters, and vice versa for ρ_03_ < 0. The change is proportional to  and can become significant for high-resolution data.

(iv) Rotation axis offset (tangential only, *n* = 0, *m* = 1),

(v) Spiral distortion (tangential only, *n* = 0, *m* = 3),

These two distortions lead to deformation matrices which, for small distortion amplitudes, are very close to pure rotation matrices and thus do not introduce any deformation of the cell dimensions.

(vi) Elliptical distortion (*n* = 2, *m* = 1, τ_21_ = ρ_21_, φ_*t*2_ =  ),

This matrix equals the unit matrix only in the (unrealistic) case of  and  . In all other cases it introduces an appreciable error. An example with α_max_ = 60°, ρ_21_ = 0.2% and  yields

This matrix introduces an error of 0.268° between vectors (1 0 0) and (0 1 0). It also introduces a difference of 0.0066 in the length of the Cartesian vectors (1 1 0) and (1 −1 0), *i.e.* a difference of 0.46%.

### b4. Derivation of matrix **L** – case of two crystals

A derivation for a general case of two crystals would be complicated. For the sake of illustrating the effect we give here the result for the particular case of two crystals rotated with respect to each other by 90°around **z**, *i.e.* the rotation matrices **R**_1_ = **I** and

Solving equation (46) for **L** yields these general expressions for the elements of **L**:

Some consequences of this result are discussed in the main text, Section 4.2.2 and Fig. 11 ▸. Here we note just that the matrix **L** does not go to the unit matrix in this case, not even for pure elliptical distortion. Thus, the combination of more crystals does not, without further measures, warrant an unbiased determination of the lattice parameters if distortions are not refined.

### b5. Derivation of matrix **L** – case with symmetry constraints

Frequently the crystal system of the investigated crystal is known or can be reasonably estimated. In that case the matrix **L** can be constrained to comply with the symmetry restrictions. We will assume the case of one crystal here for simplicity, and drop the subscript *c*.

Let us assume that the symmetry restrictions can be expressed as a set of functions *v_i_*, *i* = 1…*N_c_*, in the form *v_i_* (**L**) = 0. We may then use the method of indeterminate Lagrange multipliers to obtain the constrained minimum of *S*,

Here, λ_*i*_ needs to be set so that the conditions *v_i_* (**L**) = 0 are fulfilled.

Using the results for unconstrained minimization of *S*, we can write directly

The functions *v_i_* can be conveniently defined using the properties of the metric tensor **G**. The crystal system imposes restrictions on the elements of **G**. For example, a restriction of the unit-cell angle α to 90° is equivalent to setting *g*_23_ = 0.

Using matrix notation, an element *g_ij_* can be obtained as  , where **δ**_*i*_ is a vector with 1 at the *i*th position and 0 elsewhere. As the metric tensor can be obtained from the orientation matrix **U** as **G** = **U** ^T^ **U**, we get

As the elements of Δ**L** are typically smaller than 0.01, the last term in the sum can be neglected with little impact on the accuracy.

The functions *v_i_* (**L**) are of two types. Angle restraints to an angle α_restr_ are expressed as *v*(**L**) = *g_ij_* − (*g_ii_g_jj_*)^1/2^cosα_restr_ = 0, with a much simpler version *v*(**L**) = *g_ij_* = 0 for α_restr_ = 90°, and restraints on the equality of two cell lengths are of the form *v*(**L**) = *g_ii_* − *g_jj_* = 0.

In both cases ∂*v*_*i*_/∂**L** can be obtained from ∂*g*_*ij*_/∂**L**. Using the linearized form of *g_ij_* we obtain

We can write the result explicitly in terms of the elements *u_ij_* of **U** as

where  is the vector of the Cartesian coordinates of the lattice basis vector  . Using this result we obtain simple expressions for ∂*v*_*i*_/∂**L** as a function of only **U**. Inserting these expressions into equation (74) we obtain a set of nine linear equations with 9 + *N_c_* unknowns. Together with *N_c_* equations of the form *v_i_* (**L**) = 0 we obtain a set of 9 + *N_c_* equations with 9 + *N_c_* unknowns, which can be directly solved for **L** and λ_*i*_, *i* = 1…*N_c_*.

### b6. Correlation between **L** and distortions

The distortions are, in general, a nonlinear function of the coordinates *x_i_*, while the deformation matrix **L** is linear. Thus, in principle, the distortion parameters and the orientation matrix **U** should be refinable simultaneously from the diffraction data. In practice, however, it turns out that the coefficients of some distortions, notably of the elliptical distortion, are strongly correlated with the elements of **L**. It is desirable to have a means of quantification of this correlation, so that it can be estimated if simultaneous refinement of the orientation matrix (and thus unit-cell parameters) and distortion coefficients is possible and reliable, or if the correlation prevents a reliable combined refinement. The latter scenario means that either the unit-cell parameters or the distortion coefficients must be known from external sources in order to refine the other one reliably.

Two values are useful to quantify the degree of correlation. The correlation can be expressed by means of the standard Pearson correlation coefficient ρ,

Furthermore, the root-mean-square deviation between **u**_d_ and **u**_*L*_ [RMSD(**L**, d)] gives an estimate of the absolute value of the differences, which can then be compared with the experimental RMSD to estimate whether the expected deviation is larger or smaller than the experimental noise. RMSD(**L**, d) can be estimated as

where *V* is the volume of reciprocal space sampled in the experiment. For a single symmetric tilt series,

If the matrix **L** is obtained as a result of unconstrained least-squares minimization, the equation  holds and the correlation coefficient attains the simple form

and

However, for the general matrix **L** the full versions of the correlation coefficient and RMSD are needed. The integral  is the simplest to evaluate. Using the same steps as for the evaluation of integral **A** [equation (49)], *i.e.* transformation into spherical coordinates, we obtain an explicit form of the integral,

Using the previously defined shortcut notation *q*_20_, *q*_02_ *etc.* [equation (49)], this can be evaluated to

This expression simplifies greatly if the matrix Δ**L** has the least-squares optimized form [equation (60)]. Then, using the matrix **D** [equation (58)], we obtain

Using the definition of **u**_d_ in terms of the radial and tangential distortions [equations (1)–(5) [Disp-formula fd3] [Disp-formula fd4] [Disp-formula fd5]], the integral for  attains a particularly simple expression,  .

Using the definition of Δ*r*, we obtain an explicit expression,

which evaluates to

The integral  is completely equivalent to  , with all ρ_*nm*_ replaced with τ_*nm*_ and all φ_*rn*_ replaced by φ_*tn*_.

What remains is the evaluation of  =  . Explicitly, this integral amounts to

This integral is closely related to the integral **B** evaluated earlier, and it is conveniently expressed in terms of matrix **D**,

Here again the expression simplifies further if the expression for the least-squares-optimized Δ**L** is used, in which case, as follows from the properties of the least-squares optimization, cov(**L**, d) =  , *i.e.*

Next, we evaluate the correlation coefficients for individual types of distortion, assuming the optimal Δ**L**. This evaluation provides a good estimation of the ease or difficulty with which individual distortions can be refined together with the orientation matrix.

#### b6.1. Radial linear distortion: radial only, *n* = 0, *m* = 1

Thus ρ(**L**, d) = σ_*L*_/σ_d_ = 1 and RMSD(**L**, d) = 0, confirming the trivial result that an overall scaling distortion is perfectly correlated with scaling of the lattice.

#### b6.2. Barrel-pincushion distortion: radial only, *n* = 0, *m* = 3

Thus, regardless of the resolution and maximum tilt angle, the correlation between the barrel-pincushion distortion and lattice distortion is ∼95.8%. Although apparently high, this correlation is still sufficiently low to allow a stable refinement of the barrel-pincushion distortion in most practical cases. For a realistic value of ρ_03_ = 0.2% and  = 1.4 Å^−1^, RMSD(**L**, d) = 0.00090 Å^−1^. This value is comparable with the experimental RMSD (0.00318 Å^−1^ for DS1) and such distortion can thus be easily refined.

#### b6.3. Tangential first-order distortion (rotation axis mis­alignment): tangential only, *n* = 0, *m* = 1

The correlation tends to 1 for small α_max_, but remains very high up to relatively high α_max_. For α_max_ = 60° it is still 98.4%. RMSD(**L**, d) = 0.000054 Å^−1^ for τ_01_ = 0.0278%, which corresponds to a rotation misalignment of 0.1°. It is thus in principle possible, but in practice rather difficult, to refine the rotation misalignment accurately with this approach.

#### b6.4. Spiral distortion: tangential only, *n* = 0, *m* = 3

Although very similar to the expression for the rotation misalignment, the correlation for the spiral distortion starts at 95.8% for small α_max_ and decreases further upon increasing α_max_. The spiral distortion is thus the most robustly refinable distortion of all the standard distortions.

#### b6.5. Elliptical: *n* = 2, *m* = 1, τ_21_ = ρ_21_, φ_*t*2_ = φ_*r*2_ − π/4

This correlation coefficient is very high for all realistic values of α_max_ and it is exactly 1 for φ_*r*2_ = 0. Therefore, elliptical distortion cannot be refined together with refinement of the orientation matrix, unless symmetry constraints are applied or unless more crystals in different orientations are used.
