# Supplementary material for: Accurate lattice parameters from 3D electron diffraction data. I. Optical distortions
Source: IUCrJ. 2022 Sep 27;9(Pt 6):735–55. doi: 10.1107/S2052252522007904 (PMC9634609; doi:10.1107/S2052252522007904)

# IUCrJ

**Volume 9 (2022)**


**Supporting information for article:**

## Accurate lattice parameters from 3D electron diffraction data. I. Optical distortions

**Petr Brázda, Mariana Klementová, Yaşar Krysiak and Lukáš Palatinus**

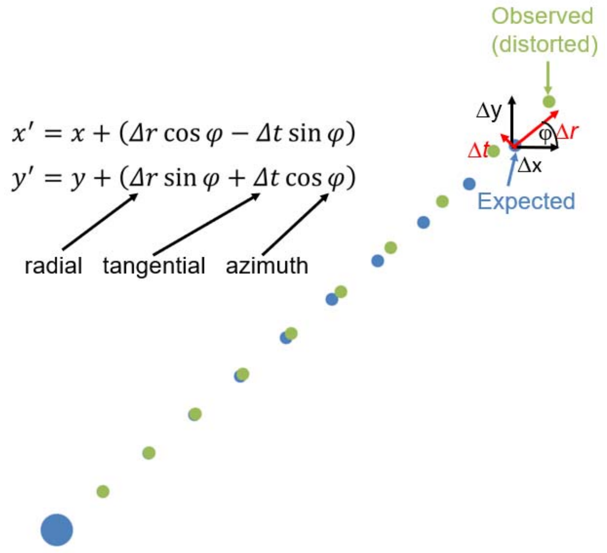


**Figure S1** Decomposition of the error between expected and observed positions of the diffraction maxima into the radial and tangential part.

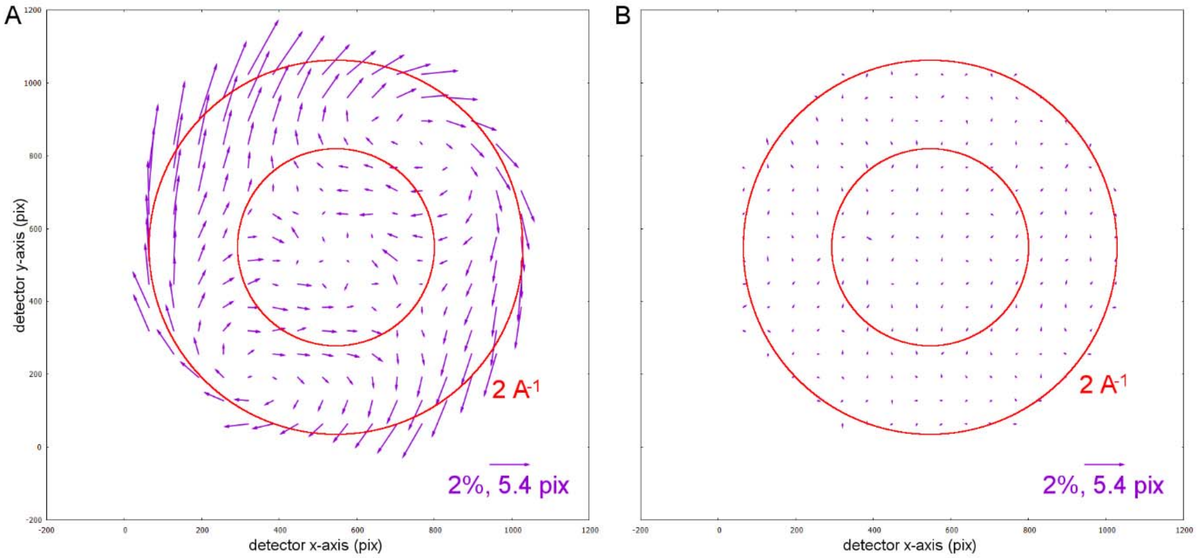


**Figure S2** Averaged differences between observed and expected positions of the diffraction maxima in dataset DS1. (A) no optical distortions were refined and the unit cell was refined with "cubic" constraint. This resulted in lattice parameter *a* equal to 11.875(1) Å. If the lattice parameters would be fixed to 11.9084 Å, the deformations would be larger. The figure is dominated by the spiral distortion. The maximum length of the average residual error was 3.15% (8.52 pix). (B) averaged residual differences after the refinement of the optical distortions, frame orientation (see Part II) and center of distortions (see Part II). No further distortion with significant amplitude can be found in the remaining residuals. The maximum length of the average residual error was 0.20% (0.55 pix).

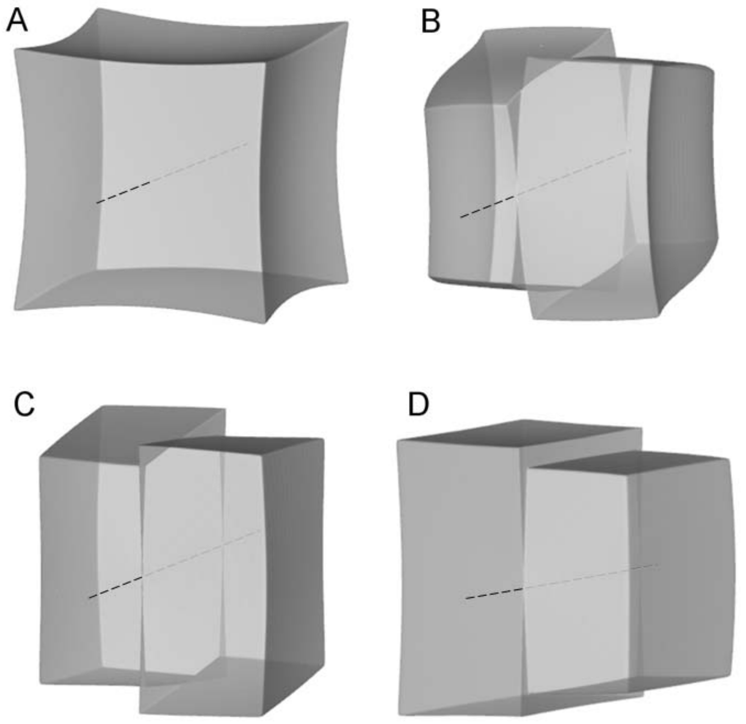


**Figure S3** Effect of 10% barrel-pincushion (A), spiral (B), elliptical (C), and parabolic (D) distortion on the deformation of the reciprocal space for tilt series measured from -90 to 90°. The shape before deformation is a cube with the center in the origin and with side of 2 $Å^{-1}$. The goniometer axis is marked by the dashed lines.

### S1. Dataset labelling

We labelled the nine datasets used in this work DS1 to DS9, and refer to them as such in the text. An overview of the datasets and their main characteristics are given in Table S1.

**Table S1** Overview of datasets used in this work

| | Tilt range (°) | Precession angle (°) | Diffraction lens excitation relative to the eucentric focus (%) | Maximal used resolution ($Å^{-1}$) |
|---|---|---|---|---|
| DS1 | -50 to +50 | 0 | 100.2 | 1.4 |
| DS2 | -50 to +50 | 0 | 100.2 | 1.4 |
| DS3 | -50 to +50 | 0 | 100.2 | 1.4 |
| DS4 | -50 to +50 | 0 | 119.2 | 3.0 |
| DS5 | -50 to +50 | 0 | 105.5 | 3.0 |
| DS6 | -50 to +50 | 0 | 105.5 | 3.0 |
| DS7 | -50 to +50 | 0 | 105.5 | 3.0 |
| DS8 | -50 to +42 | 0.92 | 105.5 | 2.0 |
| DS9 | -40 to +40 | 1.01 | 105.7 | 2.0 |

Datasets DS1, DS2 and DS3 represent standard datasets void of any specific problems, collected at the eucentric focus of the diffraction lens (intermediate lens 1 in JEOL microscopes) and with the crystal on the optical axis of the microscope. These datasets measured on three different crystals are used in Section 5.1 to illustrate the problem of correlation of the elliptical distortion with the lattice parameters. Dataset DS4 is collected to very high resolution and with very high excitation of the diffraction lens. It illustrates an extreme case of the distortions and it shows the emergence of the beam shift dependent distortions.

Datasets DS5-DS8 are collected on one crystal under the same alignment of the microscope. These four datasets were collected in order to analyse the influence of the specific ray path used during the precession experiment on the optical distortions. DS5 is a standard dataset, which serves as the reference of the distortions for the other three datasets. First, we want to understand the influence of beam deflection on the distortions in the static, non-beam-rocking, case. For this we collected two datasets in each with an electron beam tilted by 0.92° with the beam deflection coils (beam tilt) compensated by 0.92° with the image deflection coils (image tilt). The direction of the beam tilt with respect to the diffraction pattern was defined before compensating with image tilt, one with the tilt in

the x-z plane (phase 0°, data set DS6) and one with the tilt in the direction-z plane (phase 90°, data set DS7, see Figure 1). Dataset DS8 was collected as a standard dataset DS5 but this time with precession. These four datasets are used in Section 3.3, S2 and S3 to investigate the distortions specific to precession electron diffraction (PED). Finally, dataset DS9 is the standard dataset collected with precession and it is used in Section 5.2 to illustrate the precession related distortions.

### S2. Static electron beam tilt and image tilt (double tilt)

The second regime leading to observable parabolic distortion is the case of application of the image tilt, *i.e.* tilt of the whole pattern between the objective lens and the projector part of the microscope. This regime is not commonly used for electron diffraction, but it is used in precession electron diffraction. The investigation of this regime was motivated by the observation of very specific distortions in PED experiments (Section 3.3).

To analyse this case, we collected two datasets with a static doubly-tilted beam experimental setup, one with the tilt in the x-z plane (phase 0°, dataset DS6) and one with the tilt in the y-z plane (phase 90°, dataset DS7, see Figure 1). For reference, a dataset DS5 was collected without any beam/image tilt using the same settings on the same crystal. The DL excitation was in all three cases 105.5% of the eucentric focus. The angle of the beam and image tilt was 0.92°.

The double tilt experiments resulted in the appearance of the same type of distortions (magnification change, parabolic distortion and image tilt induced elliptical distortion) as in the beam shift experiment (Table S2 Part A). Table S2 Part B reflects the fact that the observed elliptical distortion is in fact a combination of two components. One is induced by the image tilt and the other, let's call it intrinsic, is present also in data without any image tilt. When the image-tilt induced elliptical distortion is extracted, it becomes clear that both the phase of the image-tilt induced parabolic distortion and the phase of the elliptical distortion depend on the phase of the image tilt (Table S2 Part A and B). We cannot exclude that the source of the parabolic distortion is the same in the beam shift and beam tilt modes because it is not possible to prove that the beam shift was perfectly uncoupled from the beam tilt. Therefore, the residual beam tilt may accompany the beam shift and induce the observed parabolic distortion. It is, however, clear that when the diffraction lens is at the eucentric focus, we do not see any primary beam shift or any parabolic distortion. These effects are observed when the diffraction lens is defocused and are more pronounced with the increasing amplitude of the defocus.

The parabolic distortion changes only marginally in different resolution shells of the diffraction data. Figure S4 shows this dependence for both the experiments with beam/image tilt. The changes are in

the range of about +/-5% for the amplitude value. The phase changes do not exceed 1.5° in both experiments. Similar observations were made for the resolution dependence of the elliptical distortion.

The important outcome of this experiment is that when we introduce the image tilt of the diffraction pattern, we induce distortions (parabolic and elliptical), which depend on the phase of the image tilt.

**Table S2** Comparison of experiments with no beam or image tilt, beam/image tilt by 0.92° with phase 0 and 90° and precession electron diffraction with precession angle equal to 0.92°. Part A contains refined magnification correction, parabolic and elliptical distortion. Part B shows the breakdown of the refined elliptical distortion into contributions from the image tilt and from the microscope optics, which are present there also without any image tilt and we call it intrinsic. Part C contains excitation error (Sg) dependent distortions, which arise from the parabolic distortion due to the precession movement of the tilted beam during the frame acquisition and thus they are present only in the precession ED experiment.

| Part A | Magnification correction (%) | Amplitude of parabolic distortion (%) | Phase of parabolic distortion (°) | Observed amplitude of elliptical distortion (%) | Observed phase of elliptical distortion (°) |
|---|---|---|---|---|---|
| DS5 (no tilt of the beam) | 0.003(2) | -0.211(1) | 35.6(2) | 0.104(1) | 54.7(3) |
| DS6 (beam/image tilt, phase 0°) | -0.558(3) | -1.244(1) | 10.6(1) | 0.350(1) | 88.6(1) |
| DS7 (beam/image tilt, phase 90°) | -0.617(3) | -1.263(1) | 95.6(1) | 0.344(1) | 13.7(1) |
| DS8 (precession ED) | -0.325(4) | -0.139(4) | -1.3(9) | 0.087(2) | 53.4(7) |

| Part B | Calculated amplitude of combined elliptical distortion (%) | Calculated phase of combined elliptical distortion (°) | Calculated amplitude of image tilt induced elliptical distortion (%) | Calculated phase of image tilt induced elliptical distortion (°) | Calculated amplitude of intrinsic elliptical distortion (%) | Calculated phase of intrinsic elliptical distortion (°) |
|---|---|---|---|---|---|---|

| | | | | | | |
|---|---|---|---|---|---|---|
| DS5 (no tilt of the beam) | - | - | - | - | - | - |
| DS6 (beam/image tilt, phase 0°) | 0.350(1) | 88.6(1) | 0.33* | 96* | 0.09** | 52** |
| DS7 (beam/image tilt, phase 90°) | 0.344(1) | 13.7(1) | 0.33 | 6 | 0.09 | 52 |
| DS8 (precession ED) | - | - | - | - | - | - |

| Part C | rSgPara (%) | tSgPara (%) | Phase (°) | rSgElli (%) | tSgElli (%) | Phase (°) |
|---|---|---|---|---|---|---|
| DS5 (no tilt of the beam) | - | - | - | - | - | - |
| DS6 (beam/image tilt, phase 0°) | - | - | - | - | - | - |
| DS7 (beam/image tilt, phase 90°) | - | - | - | - | - | - |
| DS8 (precession ED) | -0.837(2) | 0.104(2) | -21(1) | -0.143(5) | 0.026(11) | -10(3) |

* Amplitude was constrained to be equal for image tilt 0 and 90° and phase of image tilt with phase 90° was constrained to be -90° of the phase of the image tilt with phase 0°, which is equal to +90° for the elliptical distortion due to the 180° period; ** Amplitude and phase of the intrinsic contribution were constrained to be equal for the two different phases of the beam/image tilt.

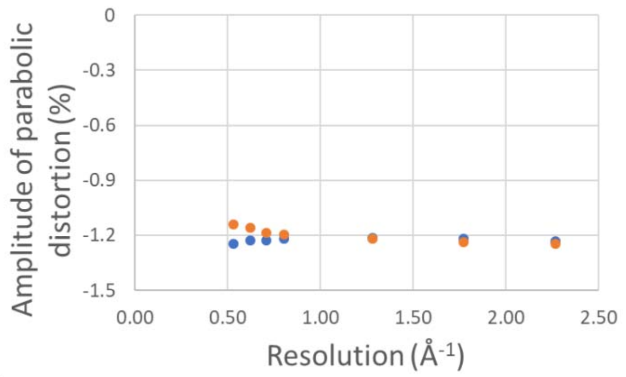


**Figure S4** Dependence of the amplitude of the parabolic distortion in different resolution shells (image tilt 0° - blue, image tilt 90° - orange). The shell thickness is 0.5 $Å^{-1}$ and its resolution is determined as volume weighted.

## S3. Discussion of the distortions relation between double tilt experiment and precession assisted data.

The precession data (DS8) were collected with the same settings and on the same crystal as the image-tilt experiments DS6 and DS7. If these experiments were a faithful representation of the two orientations of the beam during the precessing motion, we would expect the amplitudes and phases of the distortions in these experiments to be directly related to the amplitudes and phases derived from the Sg-dependent coefficients. Interestingly, this is not the case. The tSgPara coefficient is non-zero, indicating that there is a phase offset between the phase of the beam precession and the phase of the parabolic distortion. From the refined values this offset can be calculated to be -21°, contrasting with the average value of +8° in DS6 and DS7. The total amplitude of the image-tilt-induced parabolic distortion calculated from rSgPara and tSgPara is then -0.90 %. This is a smaller value than the one obtained from the datasets DS6 and DS7. The magnification correction has the same sign but it is also about a factor 1.5 smaller in DS8 than in DS6 and DS7.

SgElli coefficients representing the image tilt induced elliptical distortion (Table S2, part C) indicate that the corresponding elliptical distortion causing these coefficients has an amplitude of -0.143(5) % and phase of -10(3)° (alternatively with positive amplitude 0.143(5) % and phase 80(3)°), again smaller than the amplitude of image-tilt induced elliptical distortion observed in DS6 and DS7.

Another difference between the double tilt and precession experiments is the gradual change of the radial and tangential parts of the SgPara and SgElli coefficients in different resolution shells in DS8, which can be interpreted so that the amplitude and phase of the image tilt induced parabolic and elliptical distortions change their amplitude and phase offset as a function of reflection resolution. Figure S5 shows the evolution of these SgPara and SgElli coefficients together with the corresponding

amplitude and phase offset of the parabolic and elliptical distortions, which would cause these Sg-dependent shifts. We can see that the magnification correction remains the same for all resolutions, while the calculated image tilt induced parabolic distortion at resolution of 0.53 $Å^{-1}$ has only about half of its amplitude when compared to the maximum resolution (Figure S5B). Phase offset also changes significantly. We note, however, that this variation of the Sg-dependent coefficients with resolution, although apparently significant, does not have important practical impact on the correction of reflection positions. At low resolution, reflections attain only small excitation errors in PED. Thus, both *r* and Sg are small at low resolution, and the correction of reflection position, which is dependent on the product *r**Sg, is very small. The correction becomes appreciable only at resolution higher than approximately 1 $Å^{-1}$ (the exact magnitude of splits and shifts depends on diffraction lens excitation and precession angle), where the variation of the Sg-dependent coefficients is minor.

The overall qualitative correspondence of the results obtained on image-tilt datasets DS6 and DS7 and the precession dataset DS8 indicate the general correctness of the rationale behind the Sg-dependent coefficients. However, the quantitative differences show that the PED experiment is not exactly equivalent to a series of static beam tilt-image tilt experiments. This difference might be explained by a difference between the double tilt experiment geometry, which we have used for the static tilt experiments and the exact geometry of the precession experiment. We may also speculate that the image tilt may be also coupled with image shift. Another source of the discrepancy may be the sensitivity of the results on the exact alignment of the precession unit. The importance of this aspect is supported by the fact that the observed variation of the Sg-dependent amplitudes is not the same in various datasets, and thus depends on the details of the alignment. Despite of the discrepancies, the model for the Sg-dependent distortions developed here and implemented in PETS2 does describe the observed variation of the reflection positions very well and allows for a significantly better fit of the data collected by the precession electron diffraction method.

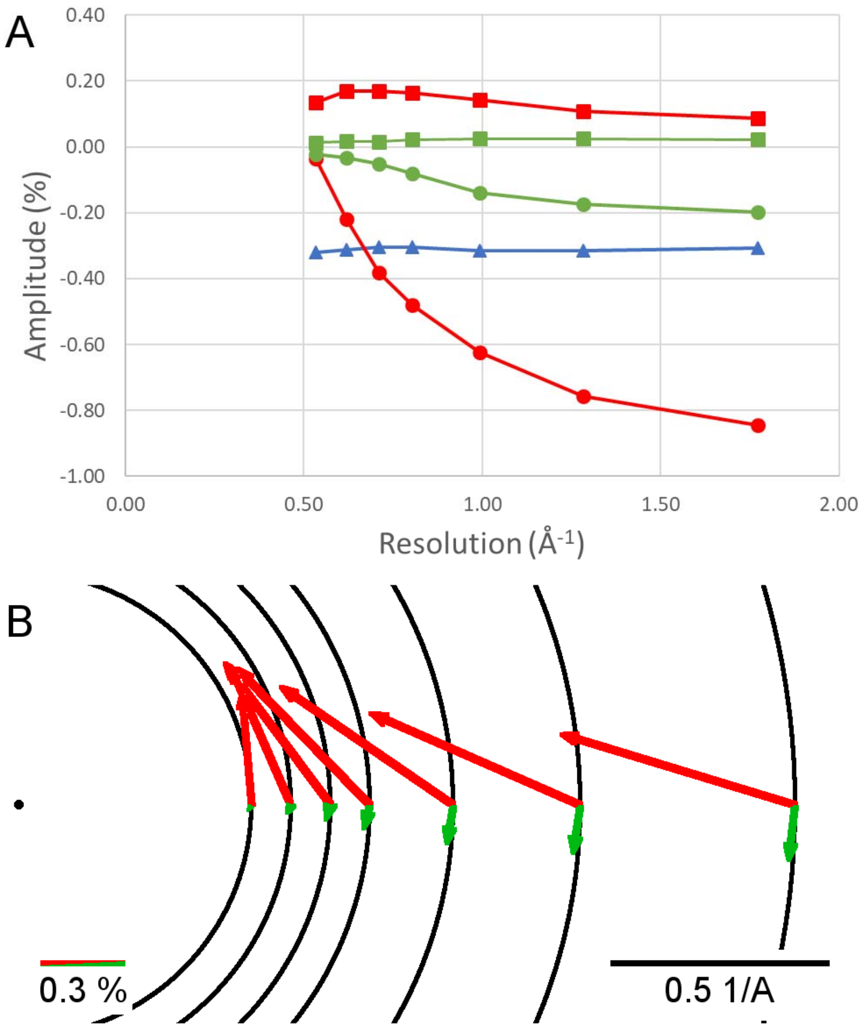


**Figure S5** Summary of resolution dependence of the precession induced distortions from the precession electron diffraction experiment (DS8) – (A) experimentally determined distortions: magnification correction (blue triangles), rSgPara (red circles), tSgPara (red squares), rSgElli (green circles), and tSgElli (green squares). (B) calculated image tilt induced parabolic (red arrows) and elliptical (green arrows) distortion amplitudes and their phases deduced from the values of the precession induced distortions (rSgPara, tSgPara, rSgElli, and tSgElli) and relations between the radial and tangential parts of the parabolic and elliptical distortions. The thickness of each resolution shell was 0.5 $Å^{-1}$ and the resolution value was determined as volume weighted resolution within the particular shells.

### S4. Examples

Two manuals for Example 1 (connected with section 5.1) and Example 2 (connected with section 5.2) are provided as supporting information. The examples provide step-by-step instructions for different approaches to optical distortion refinement implemented in the PETS2 software. They cover the problems of correlation of the elliptical distortion with the lattice parameters (Example 1) and the excitation error dependent distortions in precession electron diffraction data (Example 2). Raw data with PETS2 input files for these examples are available on ZENODO (https://doi.org/10.5281/zenodo.6424241).

#### S4.1. Example 1

## Example LuAG lattice parameters and elliptical distortion

# Input data

### Data

Electron diffraction data were measured on a transmission electron microscope FEI Tecnai $G^2$ 20 ($LaB_6$) at the accelerating voltage of 200 kV using the step-wise method (-50 to -50deg, step 1°) without precession. The sample temperature was 100 K.

### Input files

There are four folders (“DS1”, “DS2”, “DS3”, “Combined”) each containing a pts-file with the experimental information and a folder “dp-000” with 101 diffraction patterns (16-bit TIF files).

**Folders “DS1”, “DS2”, “DS3”**: individual datasets of three different crystals of LuAG (lutetium aluminum garnet)

**Folder “Combined”**: combined dataset of the three DS1, DS2, DS3 therefore 303 diffraction patterns. It contains two PETS jobs (“Combined-original.pts”, “Combined-iteration2.pts”) in which the three datasets were combined into one dataset using recalculation of the frame orientation angles of DS2 and DS3 into the reference orientation matrix of DS1.

**“Combined-original.pts”** job uses orientation matrices of DS1, DS2 and DS3, which are deformed due to the non-compensated optical distortions.

**“Combined-iteration2.pts”** job uses orientation matrices of DS1, DS2 and DS3, which are compensated for the distortion parameters of barrel-pincushion, spiral and elliptical distortions.

### Additional experimental information

Condenser lens aperture: 10 μm
Spot size: 6
Camera length: 1200 mm
Aperpix: 0.003696 $Å^{-1}.pix^{-1}$
Objective lens excitation: 89.1582% (eucentric focus 89.1582%)

Diffraction lens excitation: 36.0000% (eucentric focus 35.941%)
Calibrated distortions:

- barrel-pincushion 0.205 %
- spiral 0.461 %
- elliptical 0.394 % and 67.8°

## References

For further information about the data processing in PETS2, see:

- P. Brázda, M. Klementová, Y. Krysiak, L. Palatinus, Accurate lattice parameters from 3D electron diffraction data I: optical distortions, submitted to IUCrJ (2022).
- L. Palatinus et al. Specifics of the data processing of precession electron diffraction tomography data and their implementation in the program PETS2.0. Acta Cryst. B 75: 512-522 (2019).
- PETS2 manual pets.fzu.cz/download/

## Instructions

### 1. *Prepare (check) input file for PETS2*

*The input file for PETS is a simple text file with pts extension. In our case, the file is generated automatically during data collection. However, it can also be prepared manually after the data collection assuming all the experimental parameters are known.*

Open file 210211-07-000.pts (located in folder "DS1" in any text editor (Notepad, Notepad++, Vim, … NOT MS Word)

*Lines beginning with a hashtag are ignored by PETS. The relevant lines are the following:*

```
lambda 0.0251
Aperpixel 0.003696
dstarmax 1.4
phi 0.00
omega 0.00
center AUTO
pixelsize 0.01
noiseparameters 3.5 70
reflectionsize 10
I/sigma 5
bin 1
beamstop no
#beamstop yes T:/USERS/beamstop.xyz 950 1024
imagelist
dp-000\001.tif -50.00   0.00
…
dp-000\101.tif  50.00   0.00
endimagelist
```

***Lambda***: *relativistic wavelength in Å*
***Aperpixel:*** *size of one pixel in* $Å^{-1}$
***dstarmax:*** *resolution limit in* $Å^{-1}$ *used for data processing*
***phi:*** *precession angle in degrees*
***omega:*** *azimuth between the alpha tilt axis and positive x-axis of the image*

***center:*** *procedure for detection of primary beam*
***reflectionsize****: reflection diameter in pixels*
***bin:*** *binning factor by which the diffraction pattern resolution is reduced for processing*
***imagelist/endimagelist:*** *location of the diffraction patterns*
*Other parameters, especially concerning the detector specific noiseparameters, are explained in more detail in the manual available at http://pets.fzu.cz/*

Close the text editor.

## 2. *Run PETS2*

Start PETS by double clicking the executable or the icon.
Go to File->Open in the upper menu.
Open the file 210211-07-000.pts in the folder "DS1".

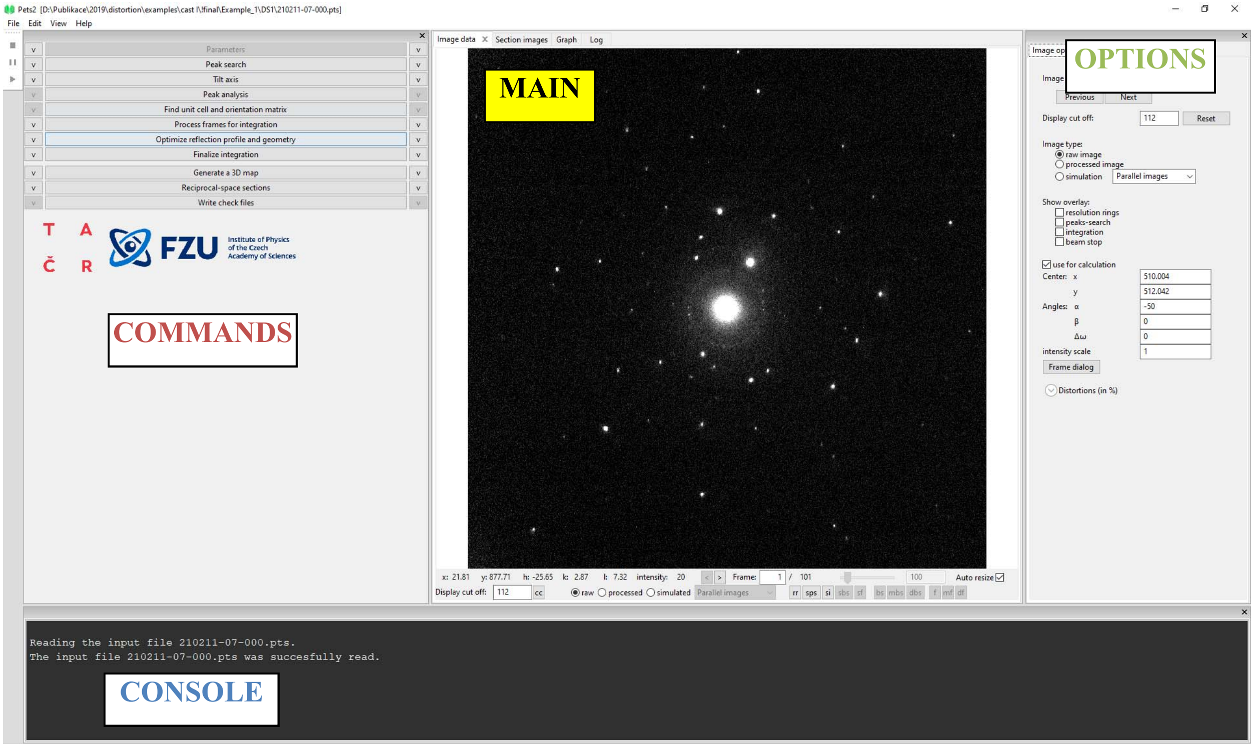


*PETS2 default view is composed of four panels:*
***Main panel*** *(in the middle) displays the data and the results of the processing.*
***Commands panel*** *(on the left) contains "**action buttons**" to trigger individual processing tasks. Parameters of the individual processing steps can be displayed (and modified) by clicking the down-arrow on the side of the action button).*
***Console*** *(at the bottom) shows detailed information about the processing (results, errors etc.).*
*Note that you can adapt the size of the different window components (e.g. make the plot window more narrow or wider) and decouple the action bar or the console from the main window.*

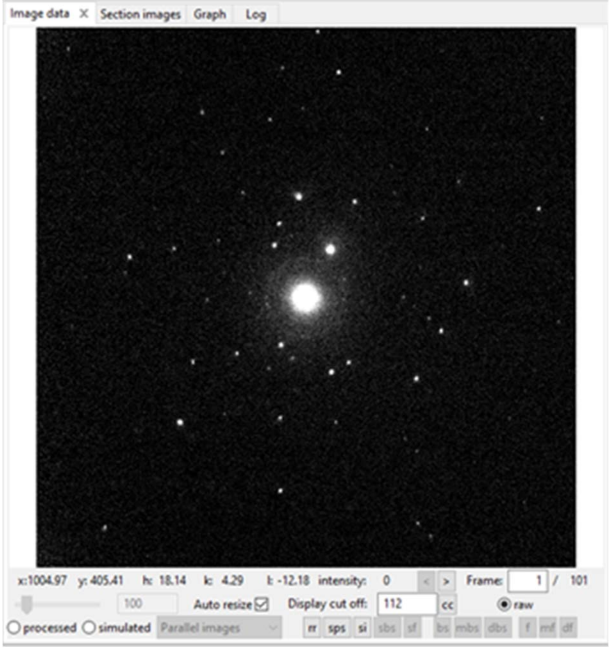


*In the Main panel (Image data tab) you can view individual diffraction images (frames) using the arrows at the bottom of the panel. The contrast of the images can be adjusted by changing the value of "Display cut off" at the bottom. For example, at 15000 only the primary beam and the strongest reflections are visible, at 10 we can clearly see the detector background noise. Values between 50 and 500 are useful to look at the relevant features, i.e. the reflections.*

## 3. *Parameters*

*Data reduction steps are run by clicking the respective action button. The action options/parameters can be modified in the panel, which opens after clicking the small arrow next to the action button. The "Parameters" action button (first in the list of action buttons) is gray because there is no associated action with it.*

Click on the arrow (v) next to "Parameters" to check the following options:

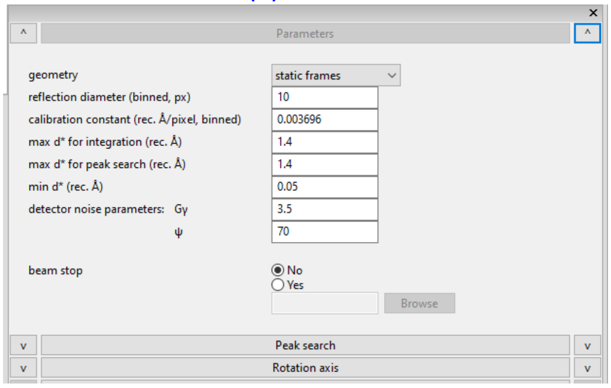


*In our input file .pts, we defined "reflectionsize 10" and "bin 1", hence the binned reflection diameter is 10 pixels. The default parameters used for min d* of 0.05 Å$^{-1}$ is OK. Here it means that any reflection/peak that is less than 0.05*Aperpixel pixel away from the primary beam is ignored. Geometry "static frames" means the dataset was measured by step-wise approach and no precession was used.*

Click on the arrow (^) next to "Parameters" to close the options.

## 4. Peak search

Click on the arrow (v) next to “Peak search” to check the following options:

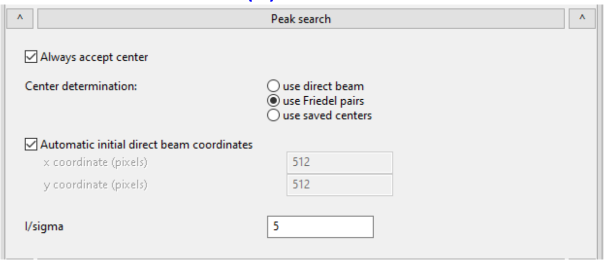


Run “Peak search” by clicking on the action button

*The console output reports that 4964 peaks were written to the raw peak list (saved as 210211-07-000.rpl in the folder “210211-07-000_petsdata”. In this folder, there are also saved nearly the all files produced by PETS except for *.cif_pets, *.hkl, *.pets2 and *_dyn.cif_pets). During peak search the pixel coordinates of the center of each diffraction pattern is determined and saved as 210211-07-000.cenloc.*

Click on “Graph” tab in the Main panel.

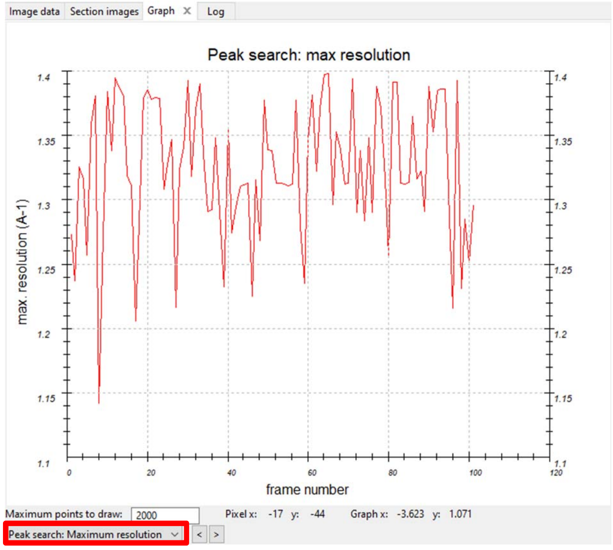


*The maximum resolution on each frame is plotted in the first graph. Deterioration of crystallinity during the experiment could be revealed here. There seems to be no problem in this dataset.*

Switch to the “Peak search: pattern centers” graph at the bottom of the panel.

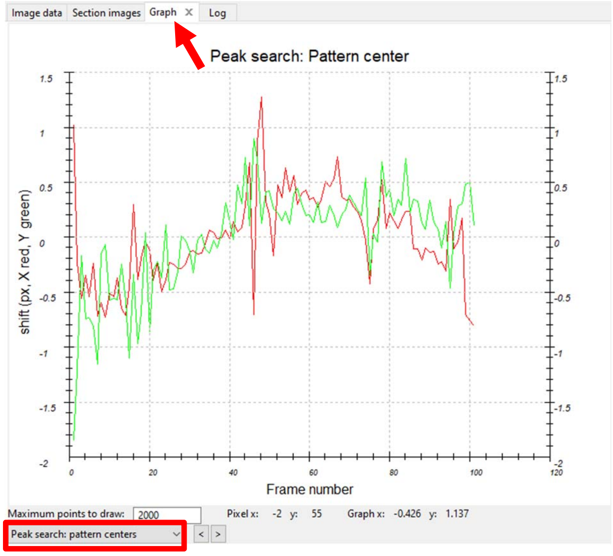


*Deviation of the center from its initial value in pixels as a function of the frame number is shown (red – x–coordinate, green – y coordinate). The shift of the primary beam is within a few pixels, which is expected for a well aligned microscope and eucentric focus of the diffraction lens. At the current stage, we assume that all pattern centers were correctly determined.*

Click on the arrow (^) next to “Peak search” to close the options.

## 5. *Tilt axis (aka rotation axis or omega)*

*The next step after peak hunting is the refinement of the angle between the horizontal axis (x axis of the diffraction image) and the projection of the tilt axis. This angle depends on the experimental setup, and needs to be refined for each data set.*

Click on the arrow (v) next to “Tilt axis” to open the options, leave all settings default.

Click the action button “Tilt axis” to start the procedure.

*In the tab “Cylindric projection” of the main panel, the difference space of the extracted peak positions is represented as a cylindric projection. In the ideal case, it consists of well-defined spots on sinusoidal curves and no elongated features. This step provides a first estimation of data quality. Here, the starting angle (defined in the input file) is 0° and it refines to 0.8230.*

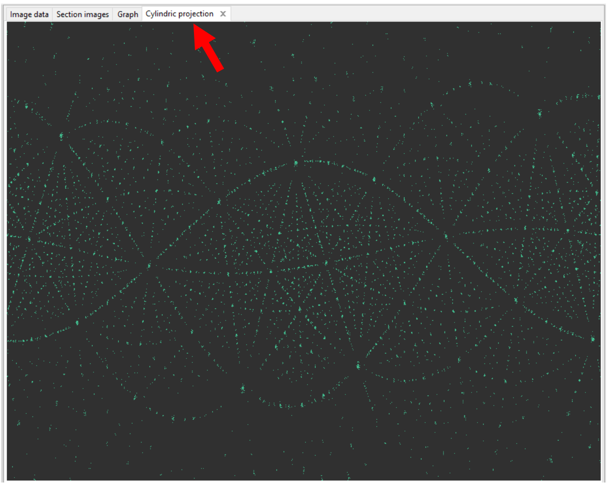


Click on the arrow (^) next to “Tilt axis” to close the options.

## 6. Peak analysis

Click the action button “Peak analysis”.

*PETS starts the analysis of the peak positions obtained in the peak hunting procedure. In the first step, distance distribution between peaks in the image plane is analysed. A sorted plot of inter-peak distances (left screenshot) is displayed in the “Graph” tab of the main panel (red curve) with its derivative (green curve).*

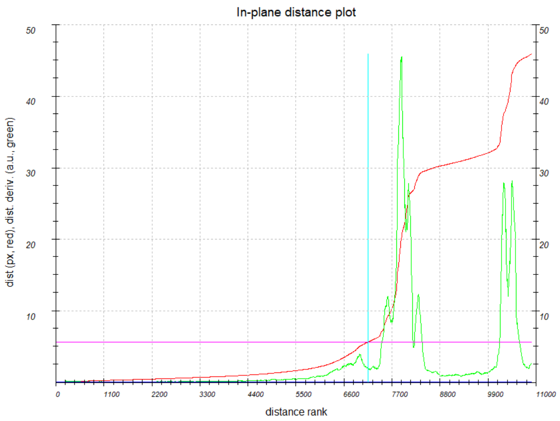


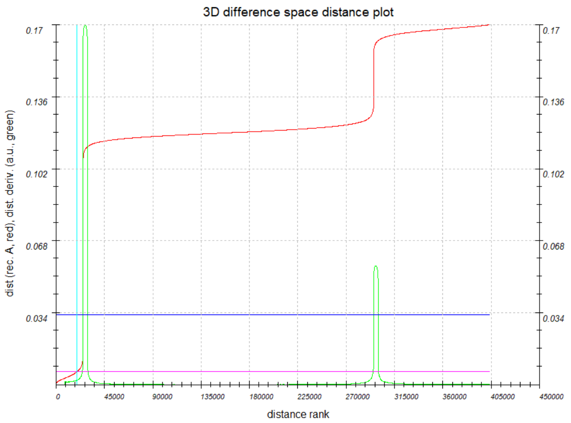


*For good quality dataset, the red curve contains distinct steps, and, consequently, the green curve has distinct peaks.*

Click “Peak analysis (continue)”.

*In the next step, an autoconvolution of the diffraction pattern (difference space) is analysed, and the groups of peaks in the autoconvolution (clusters) are replaced by the cluster centers. Again, a distance distribution is displayed (right screenshot), and clear-cut steps on the curve indicate a well-defined lattice. Peaks belonging to one reflection recorded on more frames are merged into one point and saved as 210211-07-000.xyz. Difference peaks are saved to 210211-07-000.diff.*

Click “Peak analysis (continue)”.

*Cluster map is written to 210211-07-000.clust. Now the first step is finished and PETS prepared the files needed for the determination of the orientation matrix and the unit cell parameters. At the end of this step, you should get the following message in the console:*

```
Thresholds for considering two peaks the same: 0.0053 reciprocal angstroms.
12610 peaks merged into 4940 clusters, 2770 with more than one peak.
Peak analysis successfully finished.
```

## 7. *Find unit cell and orientation matrix*

Click the action button “Find unit cell and orientation matrix”.

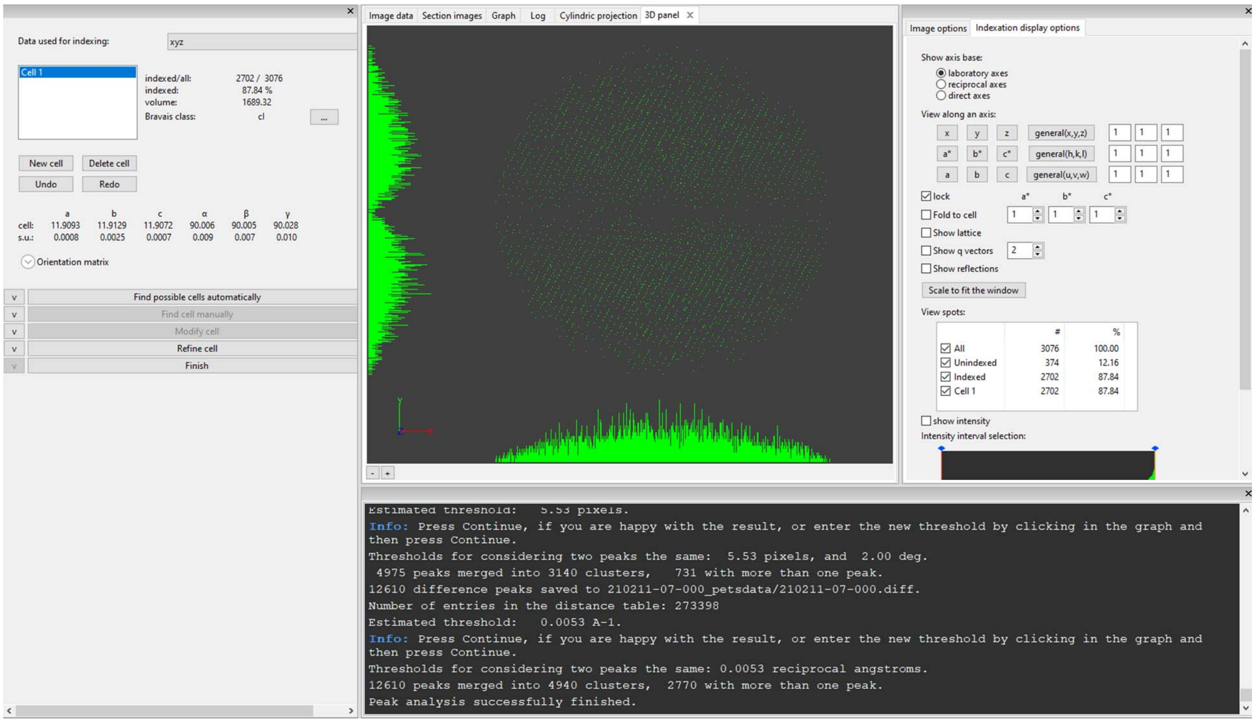


*The command panel on the left is filled by a new set of buttons to determine, refine and modify the orientation matrix, which in turn defines the unit cell parameters. In the main panel, a new tab “3D panel” opens, which it displays a projection of the peaks determined in the peak search and peak analysis. Note that the view can be enlarged by scrolling the wheel of your mouse, and rotated by holding the left mouse button. By default, the “Data used for indexing” is “xyz”, i.e. the points in 210211-07-000.xyz are used for the plot and for the orientation matrix determination.*

Click on the arrow (v) next to “Find possible cells automatically” to open the options, leave all settings default.

Click “Find possible cells automatically”.

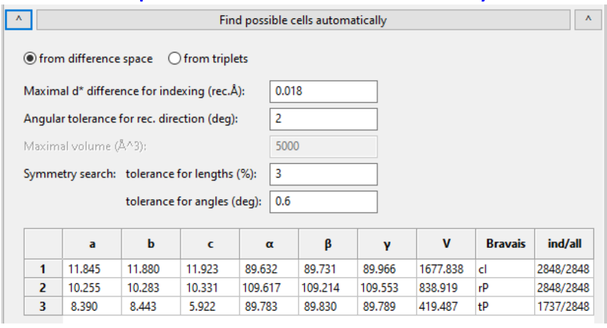


| | a | b | c | α | β | γ | V | Bravais | ind/all |
|---|---|---|---|---|---|---|---|---|---|
| 1 | 11.845 | 11.880 | 11.923 | 89.632 | 89.731 | 89.966 | 1677.838 | cI | 2848/2848 |
| 2 | 10.255 | 10.283 | 10.331 | 109.617 | 109.214 | 109.553 | 838.919 | rP | 2848/2848 |
| 3 | 8.390 | 8.443 | 5.922 | 89.783 | 89.830 | 89.789 | 419.487 | tP | 1737/2848 |

*You see the parameters used in the procedure as well as the possible unit cells found by PETS. The first one in the list (see screenshot) is automatically used. The cell angles are close to 90 degrees, a, b and c are almost equal. Therefore, PETS determined the Bravais class as cubic. To check for the possible lattice centering, in the “Indexation display options” in the options panel fold the cell to 2x2x2 and observe the I-centering (“F-centering” of the 2x2x2 folded reciprocal cell).*

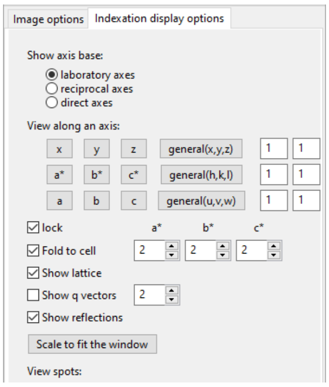

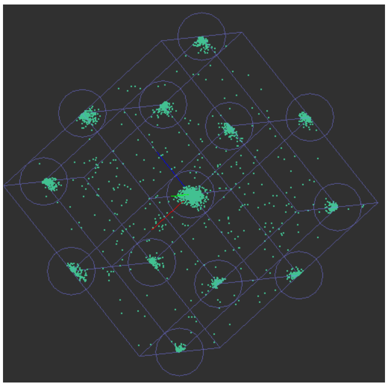

*The complete Bravais class of the unit cell is therefore cubic body-centered (cI).*
*As a part of the automatic indexing procedure the lattice parameters and orientation matrix are refined.*
Click on the arrow (^) next to “Find possible cells automatically” to close the options.
*The automatic unit cell search procedure found a unit cell, which is different from that used in the reference article (Brazda et al. 2022). To preserve the compatibility with the results given in the paper, the unit cell needs to be transformed in “Modify cell”.*
Click on the arrow (v) next to “Modify cell” to open the options.
Change the transformation matrix according to the screenshot below and press “Transform by matrix”.

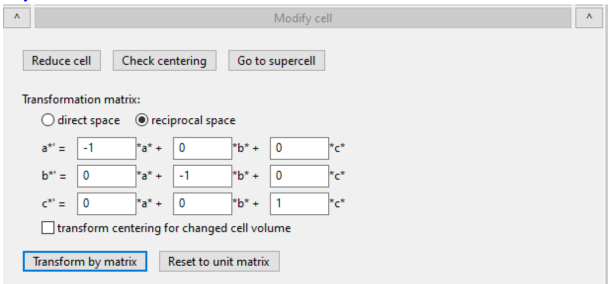


Click on the arrow (^) next to “Modify cell” to close the options.
Click on the arrow (v) next to “Refine cell” to open the options.
Choose the option “refine cell and distortions” using radio button.
Change “Maximal d* difference for indexing (Å$^{-1}$)” to 0.01.
Check only “refine cell” option.
Click on the arrow (v) next to “Distortions” to open the options.
Check that the radio button under the “^ Distortions” is set to show the distortion values in % and the tab “Standard mode” is active. Leave all the other settings as default.

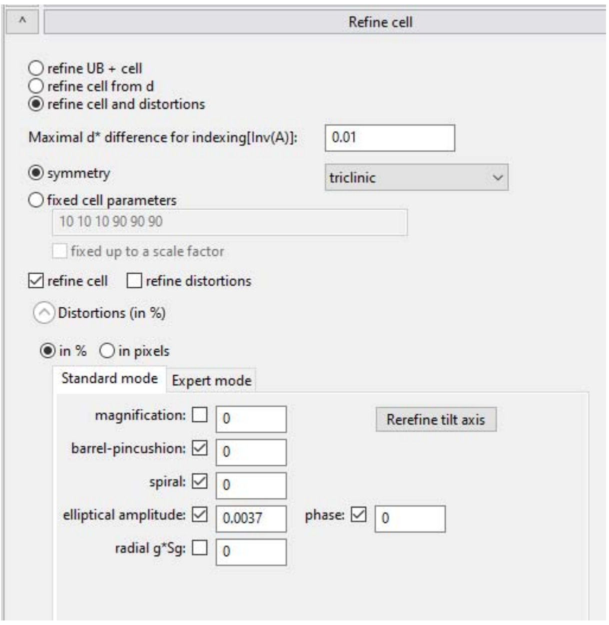


*The "Maximal d* difference for indexing" helps filter out the noise peaks from the unit cell/distortions refinement. The high quality of this dataset allows to reduce the default value to 0.01. You can visually check how well the found peaks fit the indexation and its limit by switching on the "Show reflections" option in the "Indexation display options" when the data are fold to cell. The diameter of the circle represents the "Maximal d* difference for indexing" limit.*

*There are three options for "Refine cell", which can be selected by radio button.*

(1) *"refine UB + cell" is a least-squares refinement of the orientation matrix (UB) and unit cell, which minimizes differences between the expected and observed positions of the diffraction maxima in the 3D reconstructed reciprocal space. For this refinement it is recommended to use .xyz or .cor files. These files can be selected in the "Data used for indexing".*

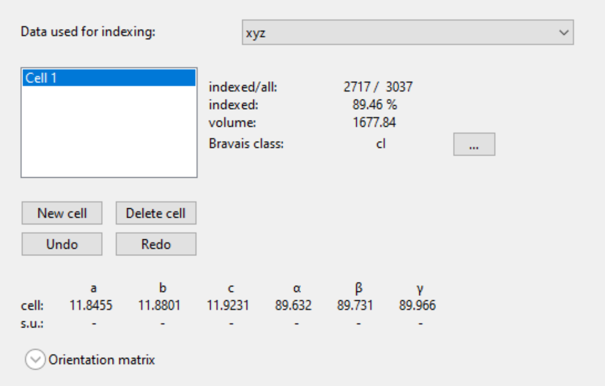


(2) *"refine cell from d" represents least-squares refinement using only interplanar spacings d (lengths of the diffraction vectors), and thus it is similar to the refinement of the unit cell in x-ray powder diffraction. 3D information about the reciprocal space is here omitted. The rotation part of the orientation matrix is determined by fixing the lattice parameters found in the refinement against d are fixed and only rotation angles are then allowed to be refined. This refinement is insensitive to the diffraction frame misorientations up to the limit when the diffraction maxima are correctly indexed. For this refinement it is recommended to use .xyz or .cor files.*

(3) *"refine cell and distortions" is least-squares refinement minimizing in-plane differences between the expected and observed positions of the diffraction maxima. Minimization is achieved through the refinement of the distortions coefficients. PETS2 automatically uses .cor data for the refinement.*

Choose "refine UB + cell" and "Data used for indexing:" .xyz. Click on "Refine cell" to proceed.

| | a | b | c | α | β | γ |
|---|---|---|---|---|---|---|
| cell: | 11.8419 | 11.8740 | 11.8993 | 90.256 | 90.293 | 89.934 |
| s.u.: | 0.0009 | 0.0017 | 0.0008 | 0.009 | 0.006 | 0.009 |

Choose "refine cell from d" and "Data used for indexing:" .xyz. Click on "Refine cell" to proceed.

| | a | b | c | α | β | γ |
|---|---|---|---|---|---|---|
| cell: | 11.8440 | 11.8561 | 11.9045 | 90.240 | 90.302 | 89.948 |
| s.u.: | 0.0000 | 0.0000 | 0.0000 | 0.000 | 0.000 | 0.000 |

Choose "refine cell and distortions". Click on "Refine cell" to proceed.

| | a | b | c | α | β | γ |
|---|---|---|---|---|---|---|
| cell: | 11.8476 | 11.8552 | 11.9068 | 90.235 | 90.324 | 89.952 |
| s.u.: | 0.0010 | 0.0030 | 0.0009 | 0.011 | 0.008 | 0.013 |

*The refinement runs for 16 cycles and the results are written in the console.*

```
   13      1.038    -0.3197    4271/   4922
   14      1.038     0.0223    4271/   4922
   15      1.038     0.0241    4271/   4922
   16      1.038    -0.0065    4271/   4922
Final orientation matrix:
  0.059115   0.030325 -0.051306
  0.054759   0.003246  0.064150
  0.025129  -0.078646 -0.017503
Cell parameters:  11.848  11.855  11.907  90.235  90.324  89.952
Finished reading   4922 peaks from the file 210211-07-000_petsdata/210211-07-000.rpl.
 4922 peaks merged into 3110 clusters,    723 with more than one peak.
12976 peaks merged into 5084 clusters,  2866 with more than one peak.
```

*The first column is the number of the cycle, which is followed the RMSD (root-mean-square deviation) between the expected and observed position of the reflection in pixels, largest change of the refined parameter expressed in fraction of its sigma, the number of peaks, which passed the "Maximal d* difference for indexing" criterion and the number of all peaks found in peak search procedure. After the refinement, the observed peak positions (including the primary beam) are corrected for the distortions using the refined distortion parameters. In this case nothing is changed, because we did not refine the distortions.*

*You can see that all the three cell refinement procedures similar results. This is because we do not have problems with frame orientation, with changing magnification, or with crystal deterioration due to its beam sensitivity but only with the optical distortions. In case there would be frame misorientations the "refine cell from d" would give better results.*
*It is generally not possible to refine freely elliptical distortion with the lattice parameters because of a strong correlation (see Section 4.1.1. in the reference article Brazda et al. 2022). This dataset is not an exception - the calculated correlation is higher than 99% and if you try to refine it both the elliptical distortion coefficients as well as the unit cell parameters diverge substantially from the expected values. We will show how to break this correlation in Section 9 of this example. In the next step, we refine only barrel-pincushion and spiral distortions.*

## 8. *Refinement of barrel-pincushion and spiral distortions and refinement of tilt axis*

*The barrel-pincushion and spiral distortions have correlation of only 0.958 and 0.943 with the orientation matrix (see Table 3 in the reference article Brazda et al. 2022). Therefore, it is possible in the decisive majority of the data to refine these distortions together with the orientation matrix (unit cell parameters). The only exceptions are data containing large inplane mosaicity or significant level of structural disorder.*

In "refine cell and distortions" enable the "refine distortions" option and check the boxes for barrel-pincushion and spiral distortions. Click on "Refine cell" to proceed.

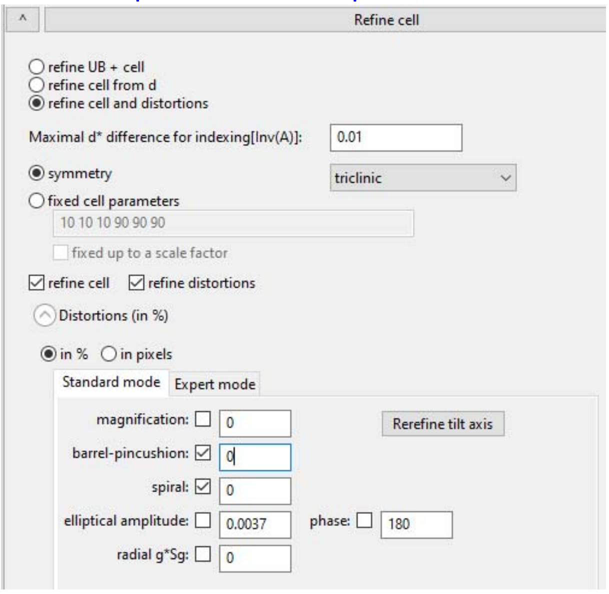


*The resulting values are 0.216 % for barrel-pincushion and 0.425 % for spiral distortion. You cannot refine magnification correction and elliptical distortion because they both correlate with unit cell parameters. The precession distortions are not present in this and the other datasets so the amplitude of rSgPara distortion would be close to zero if refined. Therefore, you should allow to refine only barrel-pincushion and spiral distortions.*

*Because this example is focused on correction of optical distortions, we will use from now on only the option "refine cell and distortions". Of course, you can check how the other refinement options work when we introduce some knowledge about the unit cell or about the distortions.*

## 9. *Breaking the correlation between lattice parameters and elliptical distortion*

*There are four options how we can break the correlation: (a) estimate the Bravais lattice symmetry, (b) use calibrated optical distortions, (c) use the lattice parameters obtained from another source like x-ray powder diffraction, and (d) combine more datasets with different orientations of the crystals towards the microscope.*

## *a. Estimated Bravais lattice symmetry*

In “Refine cell” change the “symmetry” to “monoclinic - setting “b””, check the option “refine distortions”, and enable the refinement of the barrel-pincushion, spiral and elliptical distortions.

*We guess that the Bravais lattice symmetry is monoclinic. Because the angle beta has the largest deviation from the 90°, we choose the monoclinic setting “b”.*

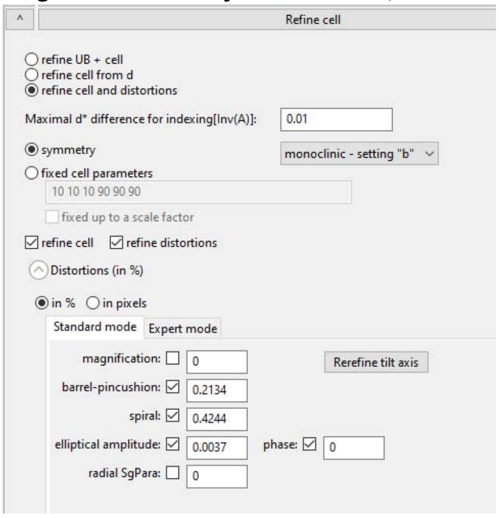


Click on “Refine cell” to proceed.

| | a | b | c | α | β | γ |
|---|---|---|---|---|---|---|
| cell: | 11.9185 | 11.9119 | 11.9023 | 90.000 | 90.025 | 90.000 |
| s.u.: | 0.0024 | 0.0023 | 0.0025 | 0.000 | 0.020 | 0.000 |

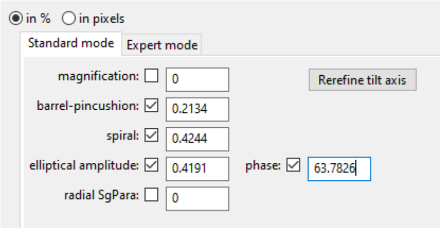


*The resulting lattice parameters are quite close to the cubic symmetry and the distortion values are also very close to the calibrated ones.*

Click on “Re-refine tilt axis”.

*The tilt axis has to be refined again because the spiral distortion deforms the diffraction frames and thus influences the global omega angle (in-plane rotation of the frame). The optimized position of the tilt axis is 0.461, thus nearly 0.4° away from the value obtained on the uncorrected data.*

Run “Refine cell” again.

*PETS runs again the “refine cell and distortions”. The unit cell improves and the distortion coefficients change slightly.*

| | a | b | c | α | β | γ |
|---|---|---|---|---|---|---|
| cell: | 11.9175 | 11.9127 | 11.9027 | 90.000 | 90.011 | 90.000 |
| s.u.: | 0.0023 | 0.0023 | 0.0025 | 0.000 | 0.019 | 0.000 |

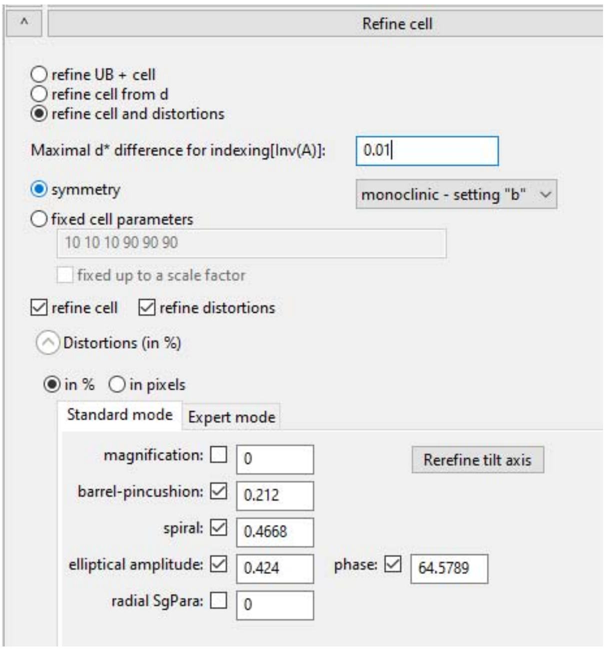


Uncheck the “refine distortions” and change the “symmetry“ to triclinic.
Press “Refine cell” to proceed.

*This is the final test of how well the data match the monoclinic unit cell. We fix the distortions and refine the unit cell without symmetry restrictions.*

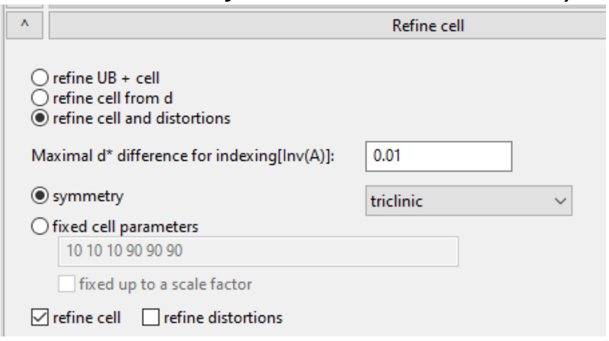


| | a | b | c | α | β | γ |
|---|---|---|---|---|---|---|
| cell: | 11.9176 | 11.9132 | 11.9027 | 89.999 | 90.012 | 90.003 |
| s.u.: | 0.0008 | 0.0025 | 0.0007 | 0.009 | 0.007 | 0.011 |

*The resulting unit cell matches very well the cubic symmetry, despite the fact that the distortions were refined only with the monoclinic restrictions.*

*In the next step, we check the cubic symmetry using the same procedure as for the monoclinic setting.*

Change “symmetry” to “cubic”. Check “refine distortions” and enable to refine barrel-pincushion, spiral and amplitude and phase of the elliptical distortion.
Press “Refine cell”.
Uncheck “refine distortions” and change symmetry to “triclinic”, press “Refine cell”.

*The tilt axis position, lattice parameters and distortions changed only marginally. In this case, the mutual orientation of the crystal and the microscope system permitted to determine the optical distortions even with the monoclinic symmetry constraints.*

| | a | b | c | α | β | γ |
|---|---|---|---|---|---|---|
| cell: | 11.9114 | 11.9120 | 11.9091 | 90.027 | 89.997 | 90.021 |
| s.u.: | 0.0008 | 0.0023 | 0.0007 | 0.002 | 0.006 | 0.010 |

## *b. Calibrated optical distortions*

In “Find unit cell and orientation matrix” open the options for “Refine cell”.
Choose “refine cell and distortions”, “symmetry” triclinic, check only “refine cell”.
Open options for “Distortions” and choose display “in %”.
Put in the calibrated values of the distortions:

- barrel-pincushion 0.205 %
- spiral 0.461 %
- elliptical distortion 0.394 % and 67.8°

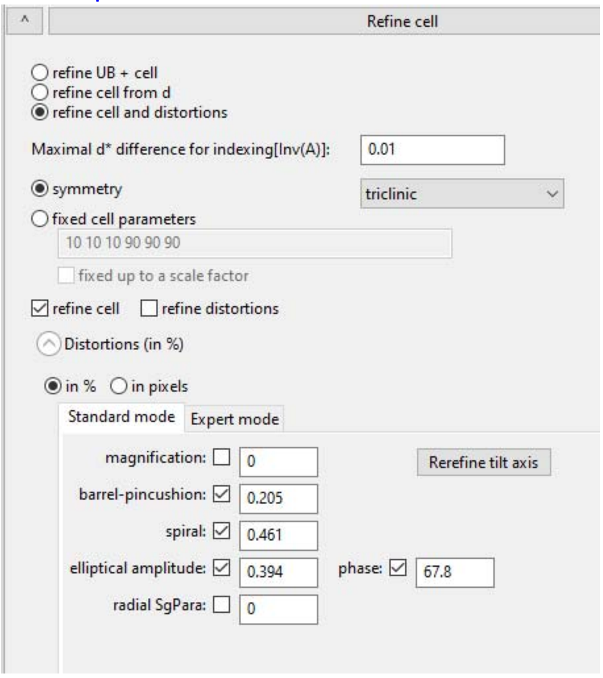


Run “Refine cell”.

| | a | b | c | α | β | γ |
|---|---|---|---|---|---|---|
| cell: | 11.9102 | 11.9096 | 11.9083 | 90.041 | 89.992 | 90.016 |
| s.u.: | 0.0008 | 0.0023 | 0.0007 | 0.002 | 0.006 | 0.010 |

*The resulting unit cell is very similar to the unit cell obtained with symmetry restrictions.*

## *c. Known lattice parameters*

*The lattice parameters were determined using x-ray powder diffraction (a = 11.9084 Å at 100 K).*
In “Find unit cell and orientation matrix” open the options for “Refine cell”.
Choose “refine cell and distortions”, choose “fixed cell parameters” and in the box below write 11.9084 11.9084 11.9084 90 90 90, check both “refine cell” and “refine distortions”.
Open options for “Distortions” and choose display “in %”.
Enable refinement of barrel-pincushion, spiral and elliptical distortion (put some small value in the amplitude in case of it is equal to zero).
Check refinement of the magnification correction.
*In this case, we keep fixed the absolute value of the known lattice parameters. Therefore, the magnification correction can be refined, which allows us to calibrate correctly the scale of the actual dataset (Aperpix value).*

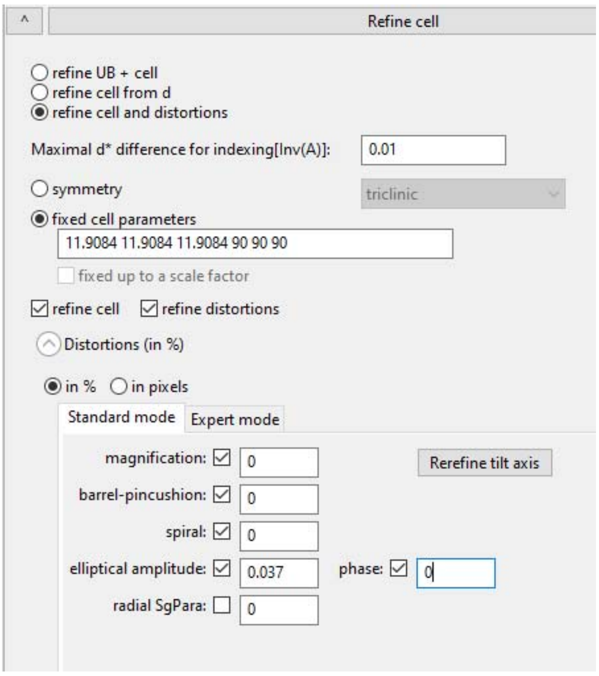


Run “Refine cell”.
Click on “Re-refine tilt axis” button.
Run “Refine cell”.
Choose “symmetry” and “triclinic”. Uncheck “refine distortions”.
*To check the stability of the result we refine freely the unit cell.*
Run “Refine cell”.
*The resulting unit cell is again very similar to the preceding ones.*

| | a | b | c | α | β | γ |
|---|---|---|---|---|---|---|
| cell: | 11.9095 | 11.9107 | 11.9072 | 90.021 | 90.000 | 90.023 |
| s.u.: | 0.0008 | 0.0023 | 0.0007 | 0.002 | 0.006 | 0.010 |

*You can see that only the alpha angle is larger by more than three sigmas than the expected values. The reason lies in fine details, which lie beyond the scope of this example and also beyond the scope of the above-mentioned reference article (Brazda et al. 2022). These details will be described in the upcoming articles.*

*You can process DS2 and DS3 in similar way we showed for DS1.*

## d. Combination of more datasets from different crystals

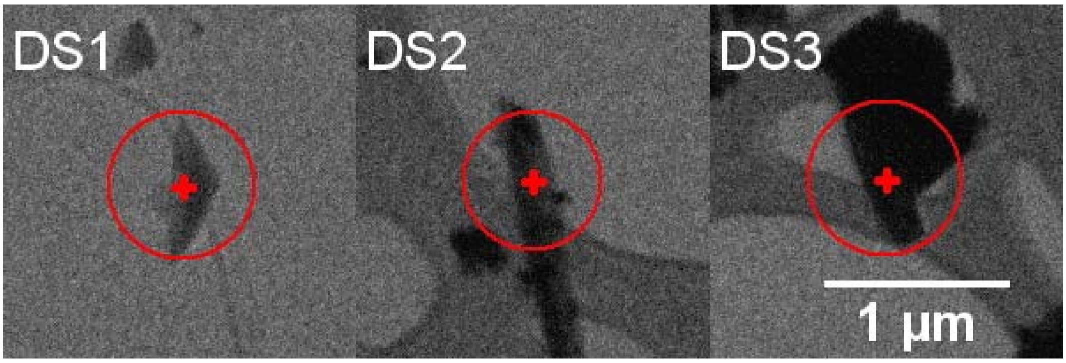


*There are three different datasets of LuAG measured under the same conditions in your data folder (DS1, DS2, DS3). It is possible to combine them into one dataset. It is possible to refine*

*the optical distortions from such a combined dataset because the orientations of the crystals (their unit cells) in the microscope are different. For dataset combination, it is necessary to know the orientation matrices of the datasets. When we do not know anything about the distortions and the lattice parameters, we know only the distorted orientation matrices. Due to this distortion, the combination suffers from incorrect frame orientation. It will be shown that only one iteration between refinement of combined dataset and refinement of the single datasets is sufficient to substantially suppress this effect.*

*Use "Combined-original" job, which was created with orientation matrices from refinement without distortions.*

Open the file Combined-original.pts.

Run "Peak search".

*15844 peaks are found in the 303 frames. The graph of the found centers of diffraction patterns indicates no problem during the procedure. Three merged datasets are clearly distinguishable.*

Click on "Graph" tab and choose "Peak search: pattern centers".

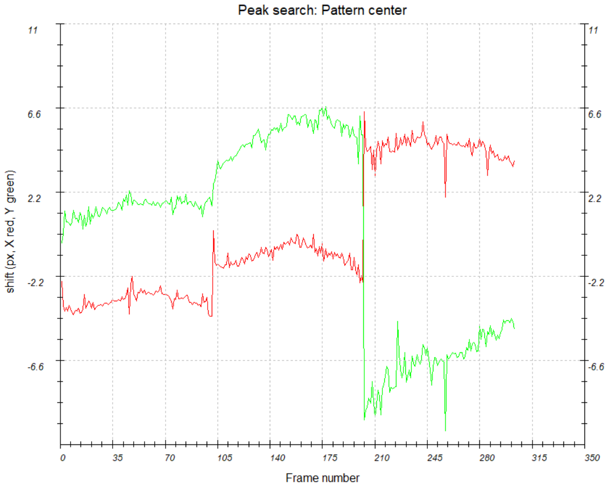


Run "Tilt axis".

*Refinement converges to 0.691°.*

Run "Peak analysis"

*Because of the combination of the three datasets, the "In-plane distance" plot is meaningless and we have to put manually the threshold to low values as it is shown in the screenshot. The 3D difference space is not significantly affected because the frame orientations are relatively correct.*

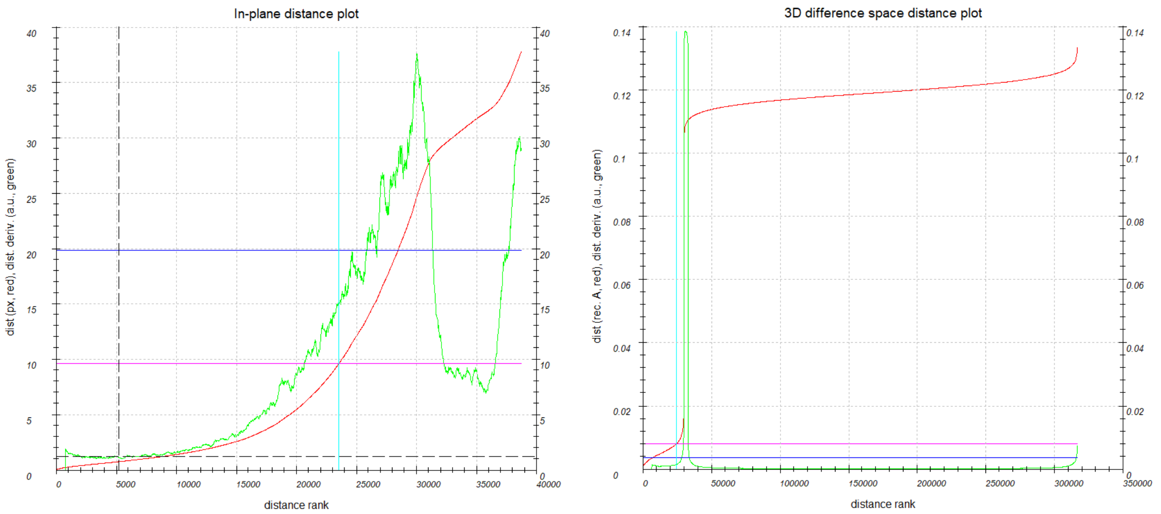


Click on “Find unit cell and orientation matrix”.
Click on the arrow (v) next to “Find possible cells automatically” to open the options, leave all settings default.
Click “Find possible cells automatically”.

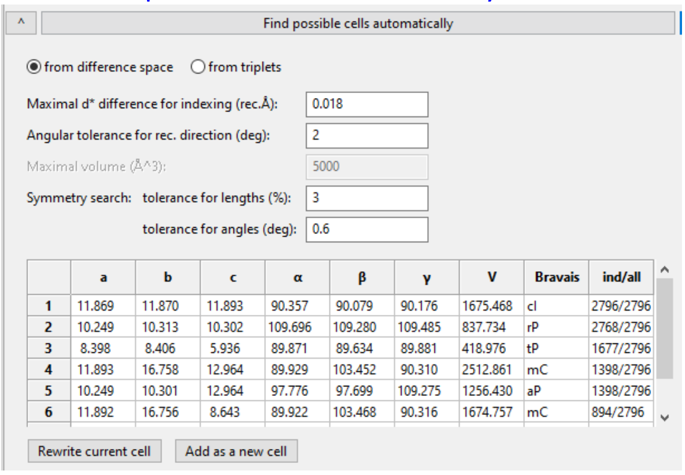


| | a | b | c | α | β | γ | V | Bravais | ind/all |
|---|---|---|---|---|---|---|---|---|---|
| 1 | 11.869 | 11.870 | 11.893 | 90.357 | 90.079 | 90.176 | 1675.468 | cI | 2796/2796 |
| 2 | 10.249 | 10.313 | 10.302 | 109.696 | 109.280 | 109.485 | 837.734 | rP | 2768/2796 |
| 3 | 8.398 | 8.406 | 5.936 | 89.871 | 89.634 | 89.881 | 418.976 | tP | 1677/2796 |
| 4 | 11.893 | 16.758 | 12.964 | 89.929 | 103.452 | 90.310 | 2512.861 | mC | 1398/2796 |
| 5 | 10.249 | 10.301 | 12.964 | 97.776 | 97.699 | 109.275 | 1256.430 | aP | 1398/2796 |
| 6 | 11.892 | 16.756 | 8.643 | 89.922 | 103.468 | 90.316 | 1674.757 | mC | 894/2796 |

Click on the arrow (^) next to “Find possible cells automatically” to close the options.
Open options for “Refine cell”. Set “maximal d* difference” to 0.01 $Å^{-1}$. Choose “refine cell and distortions”. Set “symmetry” to “triclinic”, enable both “refine cell” and “refine distortions”. Switch to display the values “in %”. Enable refinement of barrel-pincushion, spiral and elliptical distortion.

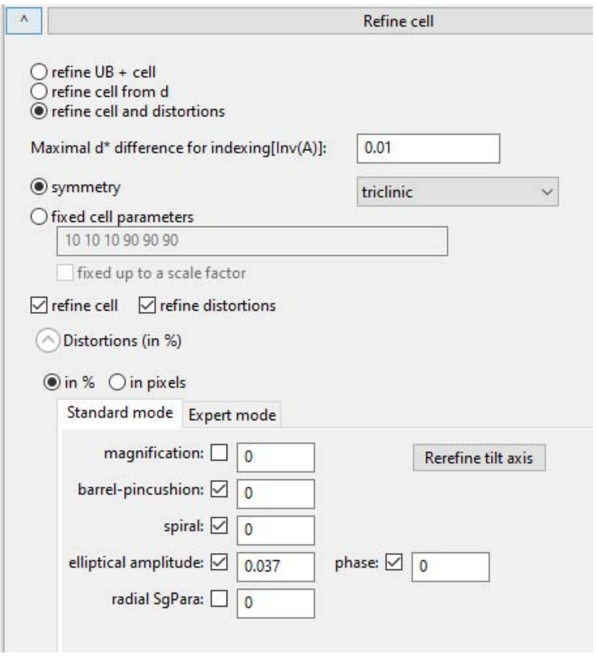


*The lattice parameters are close to cubic symmetry and the distortions are close to the calibrated ones. This proves that with this approach it is possible to refine freely the unit cell and the elliptical distortion. However, all the angles are relatively far away from the expected 90°. This is because of the problems with frame orientation, which have origins in the deformed orientation matrices of the original datasets.*

| | a | b | c | α | β | γ |
|---|---|---|---|---|---|---|
| cell: | 11.9054 | 11.8968 | 11.9057 | 90.161 | 90.080 | 90.107 |
| s.u.: | 0.0009 | 0.0013 | 0.0009 | 0.002 | 0.003 | 0.005 |

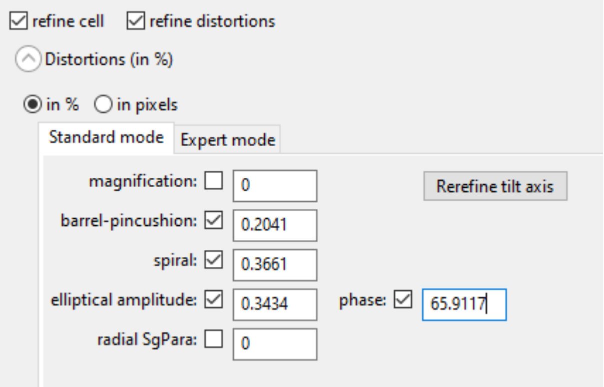


*When we use "refine cell from d" this problem is significantly suppressed.*

| | a | b | c | α | β | γ |
|---|---|---|---|---|---|---|
| cell: | 11.9017 | 11.9098 | 11.9106 | 89.977 | 90.020 | 90.037 |
| s.u.: | 0.0000 | 0.0000 | 0.0000 | 0.000 | 0.000 | 0.000 |

*We can now use the first estimation of the distortions from the combined dataset refinement to improve the orientation matrices of the original datasets. Thus, to use these distortions as we used the calibrated ones in the part 8.b.*

Click on "Finish".

*This combined dataset is prepared for you (Combined-Iteration2).*

Open the file "Combined-iteration2.pts".

Run "Peak search".

*15844 peaks are found in the 303 frames. The graph of the found centers of diffraction patterns indicates no problem during the procedure. Three merged datasets are clearly distinguishable.*

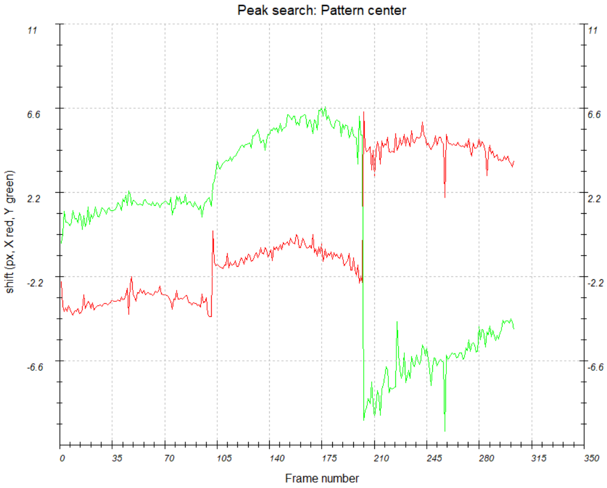


Run “Tilt axis”.

*The omega angle value converges to 0.626°.*

Run “Peak analysis”

*Because of the combination of the three datasets, the “In-plane distance” is meaningless and we have to put manually the threshold to low values as it is shown in the screenshot. The 3D difference space is not significantly affected because the frame orientations are relatively correct.*

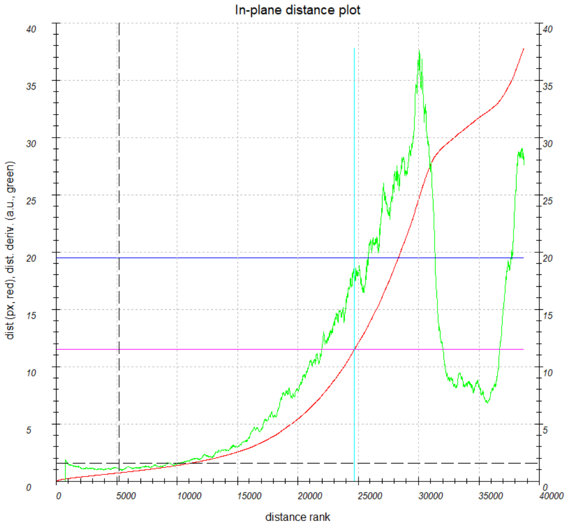


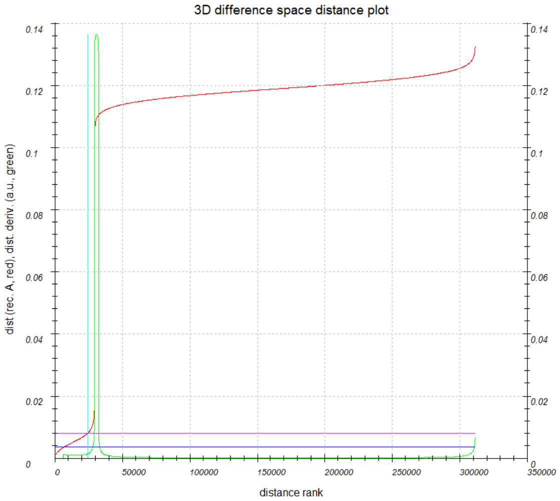


Click on “Find unit cell and orientation matrix”.

Click on the arrow (v) next to “Find possible cells automatically” to open the options, leave all settings default.

Click “Find possible cells automatically”.

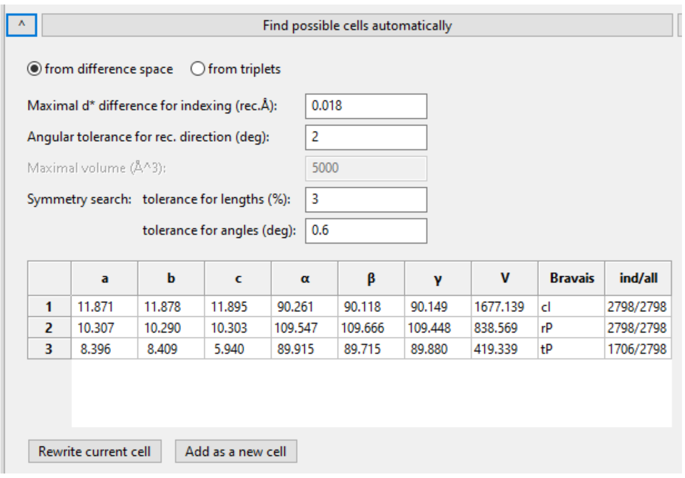


Click on the arrow (^) next to “Find possible cells automatically” to close the options. Open options for “Refine cell”. Set “maximal d* difference” to 0.01 Å$^{-1}$. Choose “refine cell and distortions”. Set “symmetry” to “triclinic”, enable both “refine cell” and “refine distortions”. Switch to display the values “in %”. Enable refinement of barrel-pincushion, spiral and elliptical distortion.
After the refinement click on the “Re-refine tilt axis” button.
Run the final “Refine cell”.

*The lattice parameters are very close to cubic symmetry and this improvement is caused by the improvement in the frame orientation. The value of the elliptical distortion is also closer to the calibrated one. Further improvements of the match between the expected and observed cell and distortions are possible, but beyond the scope of this example.*

| | a | b | c | α | β | γ |
|---|---|---|---|---|---|---|
| cell: | 11.9055 | 11.9073 | 11.9069 | 90.016 | 90.025 | 90.040 |
| s.u.: | 0.0008 | 0.0011 | 0.0008 | 0.003 | 0.003 | 0.005 |

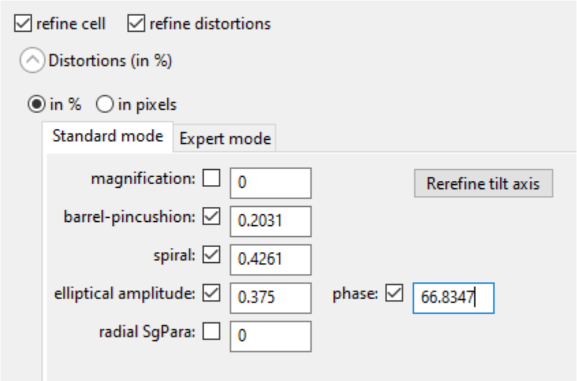


Click on “Finish”.

**S4.2. Example 2**

# Example LuAG parabolic distortion in PED data

## Input data

### Data

Electron diffraction data were measured on a transmission electron microscope FEI Tecnai G2 20 (LaB6) at the accelerating voltage of 200 kV using the step-wise method (-40 to -40deg, step 1°) with beam precession of 1°. The sample temperature was 100 K.

### Input files

PETS job file 210526-06-100.pts with the experimental information and folder “dp-100” containing 81 diffraction patterns (16-bit TIF files).

### Additional experimental information

Condenser lens aperture: 10 μm
Spot size: 6
Camera length: 1000 mm
Aperpix: 0.004673 $Å^{-1}.pix^{-1}$
Objective lens excitation: 89.2032% (eucentric focus 89.1582%)

Diffraction lens excitation: 38.0000% (eucentric focus 35.941%)
Calibrated distortions: barrel-pincushion -0.444 $\%.Å^2$, spiral 0.334 $\%.Å^2$, and elliptical 0.113 % and 60.1°.

## References

For further information about the data processing in PETS2, see:
- L. Palatinus et al. Specifics of the data processing of precession electron diffraction tomography data and their implementation in the program PETS2.0. Acta Cryst. B 75: 512-522 (2019).
- PETS2 manual pets.fzu.cz/download/

## Instructions

## 1. *Prepare (check) input file for PETS2*

*The input file for PETS is a simple text file with pts extension. In our case, the file is generated automatically during data collection. However, it can also be prepared manually after the data collection assuming all the experimental parameters are known.*
Open file 210526-06-100.pts (located in folder “DS1” in any text editor (Notepad, Notepad++, Vim, … NOT MS Word)
*Lines beginning with a hashtag are ignored by PETS. The relevant lines are the following:*

```
lambda 0.0251
Aperpixel 0.004673
dstarmax 2.0
dstarmaxps 1.4
phi 1.01
omega 0.00
center AUTO
pixelsize 0.01
noiseparameters 3.5 70
reflectionsize 10
I/sigma 5
bin 1
beamstop no
#beamstop yes T:/USERS/beamstop.xyz 950 1024
imagelist
dp-000\001.tif -40.00   0.00
…
dp-000\081.tif  40.00   0.00
endimagelist
```

***Lambda***: *relativistic wavelength in Å*

***Aperpixel:*** *size of one pixel in Å$^{-1}$*

***dstarmax:*** *resolution limit in Å$^{-1}$used for data processing*

***phi:*** *precession angle in degrees*

***omega:*** *azimuth between the alpha tilt axis and positive x-axis of the image*

***center:*** *procedure for detection of primary beam*

***reflectionsize***: *reflection diameter in pixels*

***bin:*** *binning factor by which the diffraction pattern resolution is reduced for processing*

***imagelist/endimagelist:*** *location of the diffraction patterns*

*Other parameters, especially concerning the detector specific noiseparameters, are explained in more detail in the manual available at [http://pets.fzu.cz/](http://pets.fzu.cz/)*

Close the text editor.

## 2. *Run PETS2*

Start PETS by double clicking the executable or the icon.
Go to File->Open in the upper menu
Open the file 210526-06-100.pts in the folder

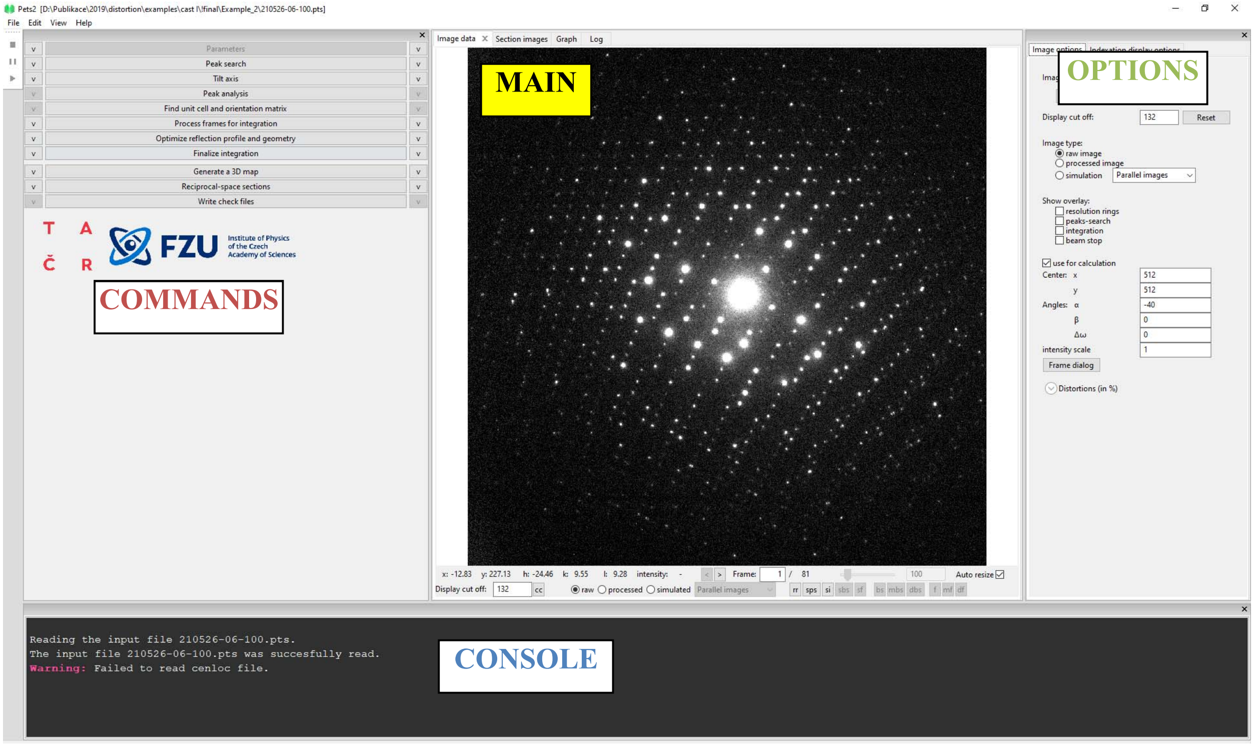


*PETS2 default view is composed of four panels:*

***Main panel** (in the middle) displays the data and the results of the processing.*

***Commands panel** (on the left) contains "**action buttons**" to trigger individual processing tasks.*

*Parameters of the individual processing steps can be displayed (and modified) by clicking the down-arrow on the side of the action button).*

***Console** (at the bottom) shows detailed information about the processing (results, errors etc.).*

*Note that you can adapt the size of the different window components (e.g. make the plot window more narrow or wider) and decouple the action bar or the console from the main window.*

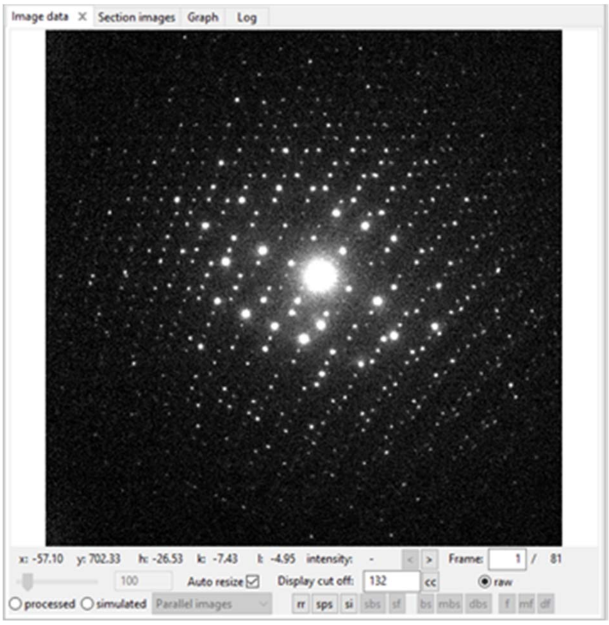


*In the Main panel (Image data tab) you can view individual diffraction images (frames) using the arrows at the bottom of the panel. The contrast of the images can be adjusted by*

*changing the value of "Display cut off" at the bottom. For example, at 15000 only the primary beam and the strongest reflections are visible, at 10 we can clearly see the detector background noise. Values between 50 and 500 are useful to look at the relevant features, i.e. the reflections.*

## 3. Parameters

*Data reduction steps are run by clicking the respective action button. The action options/parameters can be modified in the panel, which opens after clicking the small arrow next to the action button. The "Parameters" action button (first in the list of action buttons) is gray because there is no associated action with it.*

Click on the arrow (v) next to "Parameters" to check the following options:

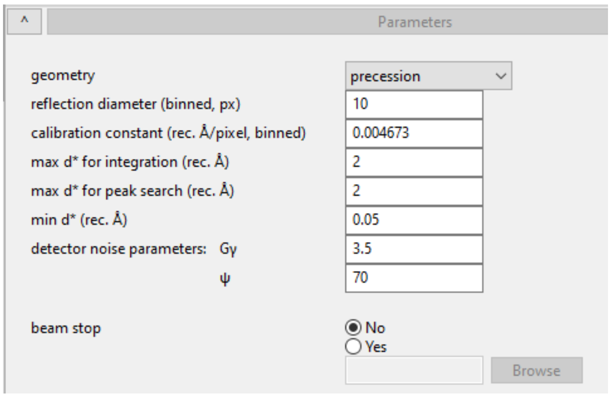


*In our input file .pts, we defined "reflectionsize 10" and "bin 1", hence the binned reflection diameter is 10 pixels. The default parameters used for min d* of 0.05 Å$^{-1}$ is OK. Here it means that any reflection/peak that is less than 0.05*Aperpixel pixel away from the primary beam is ignored. Geometry is "precession" because the dataset was measured by step-wise approach with precession.*

Click on the arrow (^) next to "Parameters" to close the options.

## 4. Peak search

Click on the arrow (v) next to "Peak search" to check the following options:

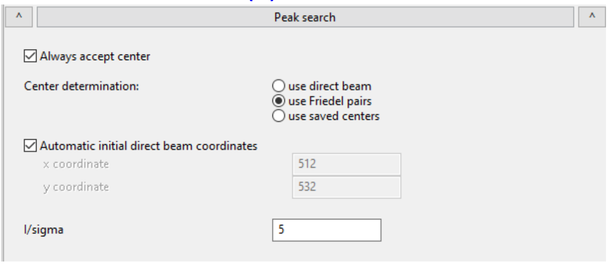


Run "Peak search" by clicking on the action button

*The console output reports that 15722 peaks were written to the raw peak list (saved as 210526-06-100.rpl in the folder "210526-06-100_petsdata". In this folder, there are also saved nearly the all files produced by PETS except for *.cif_pets, *.hkl, *.pets2 and *_dyn.cif_pets). During peak search the pixel coordinates of the center of each diffraction pattern is determined and saved as 210526-06-100.cenloc.*

Click on "Graph" tab in the Main panel.

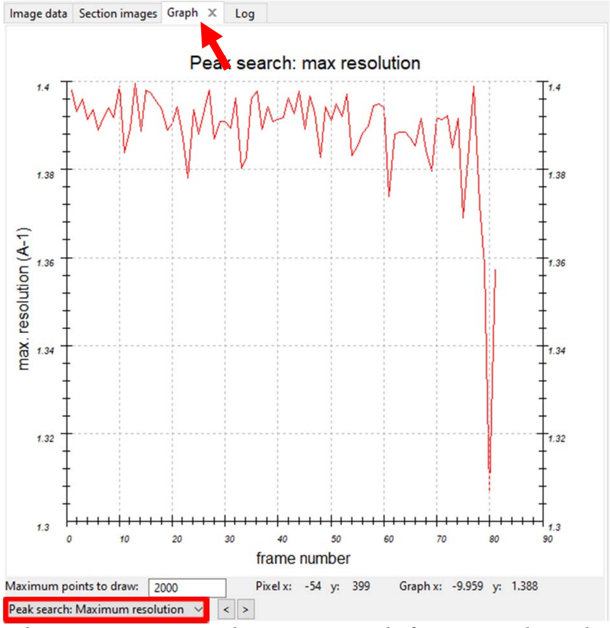


*The maximum resolution on each frame is plotted in the first graph. Deterioration of crystallinity during the experiment could be revealed here. There seems to be no problem in this dataset.*

Switch to the "Peak search: pattern centers" graph at the bottom of the panel.

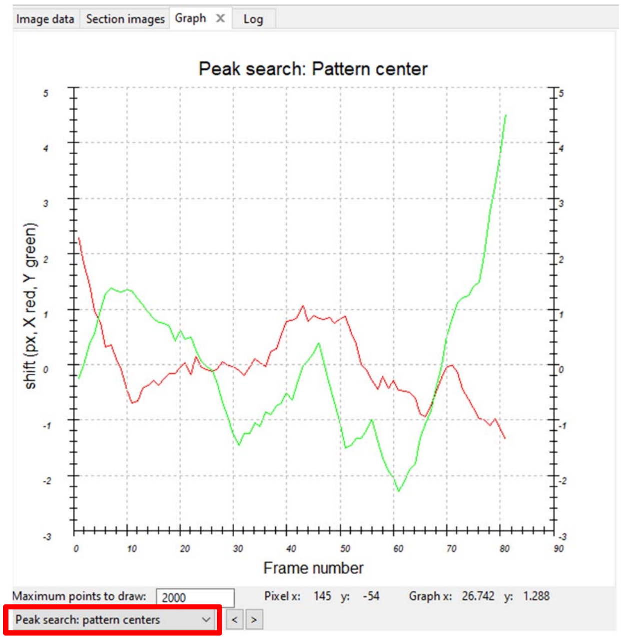


*Deviation of the center from its initial value in pixels as a function of the frame number is shown (red – x–coordinate, green – y coordinate). The shift of the primary beam is within a few pixels, which is expected for a well aligned microscope and eucentric focus of the diffraction lens. At the current stage, we assume that all pattern centers were correctly determined.*

Click on the arrow (^) next to "Peak search" to close the options.

## *5. Tilt axis (aka rotation axis or omega)*

*The next step after peak hunting is the refinement of the angle between the horizontal axis (x axis of the diffraction image) and the projection of the tilt axis. This angle depends on the experimental setup, and needs to be refined for each data set.*

Click on the arrow (v) next to "Tilt axis" to open the options, leave all settings default.

Click the action button "Tilt axis" to start the procedure.

*In the tab "Cylindric projection" of the main panel, the difference space of the extracted peak positions is represented as a cylindric projection. In the ideal case, it consists of well-defined spots on sinusoidal curves and no elongated features. This step provides a first estimation of data quality. Here, the starting angle (defined in the input file) is 0° and it refines to -2.913, which is in a good agreement with expected -2.5°.*

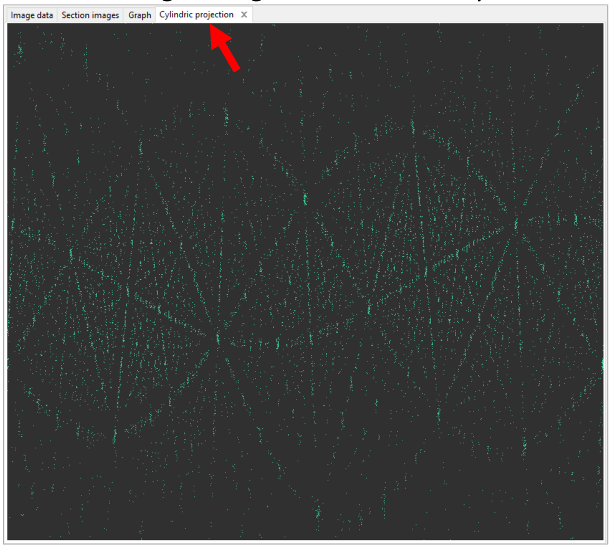


Click on the arrow (^) next to "Tilt axis" to close the options.

# 6. *Peak analysis*

Click the action button "Peak analysis".

*PETS starts the analysis of the peak positions obtained in the peak hunting procedure. In the first step, distance distribution between peaks in the image plane is analysed. A sorted plot of inter-peak distances (left screenshot) is displayed in the "Graph" tab of the main panel (red curve) with its derivative (green curve).*

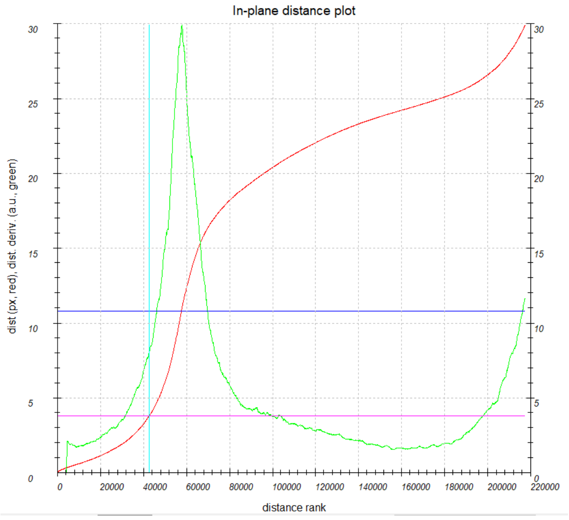


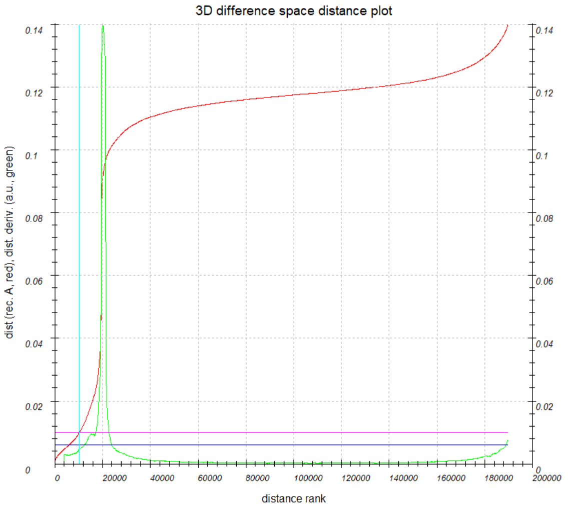


*For good quality data sets, the red curve contains distinct steps, and, consequently, the green curve has distinct peaks.*

Click "Peak analysis (continue)".

*In the next step, an autoconvolution of the diffraction pattern (difference space) is analysed, and the groups of peaks in the autoconvolution (clusters) are replaced by the cluster centers. Again, a distance distribution is displayed (right screenshot), and clear-cut steps on the curve indicate a well-defined lattice. Peaks belonging to one reflection recorded on more frames are merged into one point and saved as 210526-06-100.xyz. Difference peaks are saved to 210526-06-000.diff.*

Click "Peak analysis (continue)".

*Cluster map is written to 210526-06-100.clust. Now the first step is finished and PETS prepared the files needed for the determination of the orientation matrix and the unit cell parameters. At the end of this step, you should get the following message in the console:*

```
Thresholds for considering two peaks the same: 0.0106 reciprocal angstroms.
11384 peaks merged into 7104 clusters, 2002 with more than one peak.
Peak analysis successfully finished.
```

## 7. *Find unit cell and orientation matrix*

Click "Find unit cell and orientation matrix".

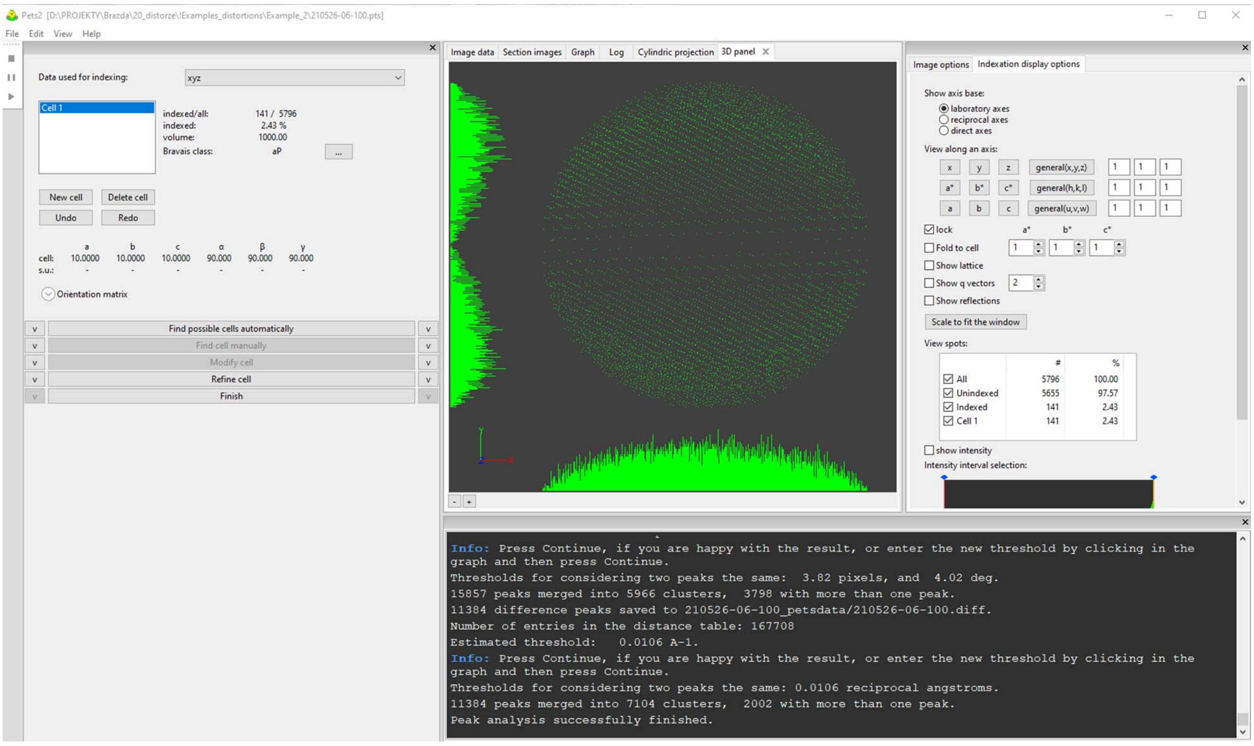


*The command panel on the left is filled by a new set of buttons to determine, refine and modify the orientation matrix, which in turn defines the unit cell parameters. In the main panel, a new tab "3D panel" opens, which it displays a projection of the peaks determined in the peak search and peak analysis. Note that the view can be enlarged by scrolling the wheel of your mouse, and rotated by holding the left mouse button. By default, the "Data used for indexing" is "xyz", i.e. the points in 210526-06-100.xyz are used for the plot and for the orientation matrix determination.*

Click on the arrow (v) next to "Find possible cells automatically" to open the options, leave all settings default.

Click "Find possible cells automatically".

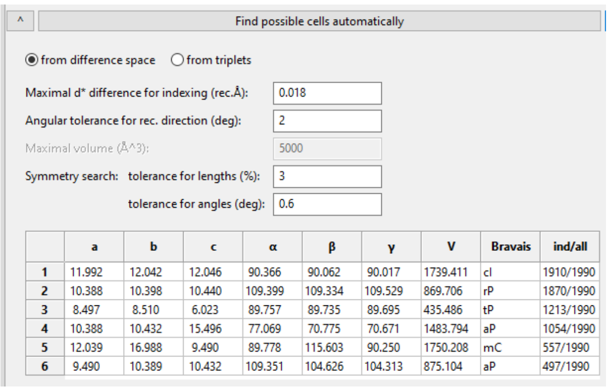


| | a | b | c | α | β | γ | V | Bravais | ind/all |
|---|---|---|---|---|---|---|---|---|---|
| 1 | 11.992 | 12.042 | 12.046 | 90.366 | 90.062 | 90.017 | 1739.411 | cI | 1910/1990 |
| 2 | 10.388 | 10.398 | 10.440 | 109.399 | 109.334 | 109.529 | 869.706 | rP | 1870/1990 |
| 3 | 8.497 | 8.510 | 6.023 | 89.757 | 89.735 | 89.695 | 435.486 | tP | 1213/1990 |
| 4 | 10.388 | 10.432 | 15.496 | 77.069 | 70.775 | 70.671 | 1483.794 | aP | 1054/1990 |
| 5 | 12.039 | 16.988 | 9.490 | 89.778 | 115.603 | 90.250 | 1750.208 | mC | 557/1990 |
| 6 | 9.490 | 10.389 | 10.432 | 109.351 | 104.626 | 104.313 | 875.104 | aP | 497/1990 |

*You see the parameters used in the procedure as well as the possible unit cells found by PETS. The first one in the list (see screenshot) is automatically used. The cell angles are close to 90 degrees, a, b and c are almost equal. Therefore, PETS determined the Bravais class as cubic. To check for the possible lattice centering, in the "Indexation display options" in the*

*options panel fold the cell to 2x2x2 and observe the I-centering (“F-centering” of the 2x2x2 folded reciprocal cell).*

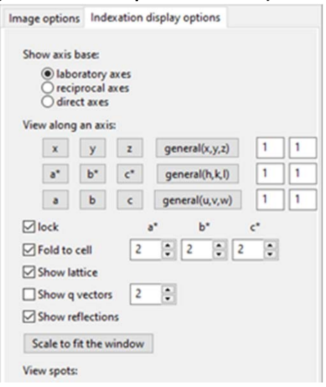

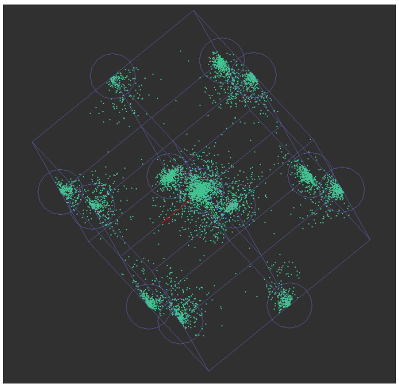

*The complete Bravais class of the unit cell is therefore cubic body-centered (cI).*
*As a part of the automatic indexing procedure the lattice parameters and orientation matrix are refined.*

Click on the arrow (^) next to “Find possible cells automatically” to close the options.
Click on the arrow (v) next to “Refine cell” to open the options.
Choose “refine cell and distortions” and set “symmetry” constraint to “cubic”.
Check “refine cell” and “refine distortions”.
Click on the arrow (v) next to “Distortions” to open the options.
Check that the radio button under the “^ Distortions” is set to show the distortion values in % and the tab “Standard mode” is active.
Enable refinement of barrel-pincushion, spiral and elliptical distortion.

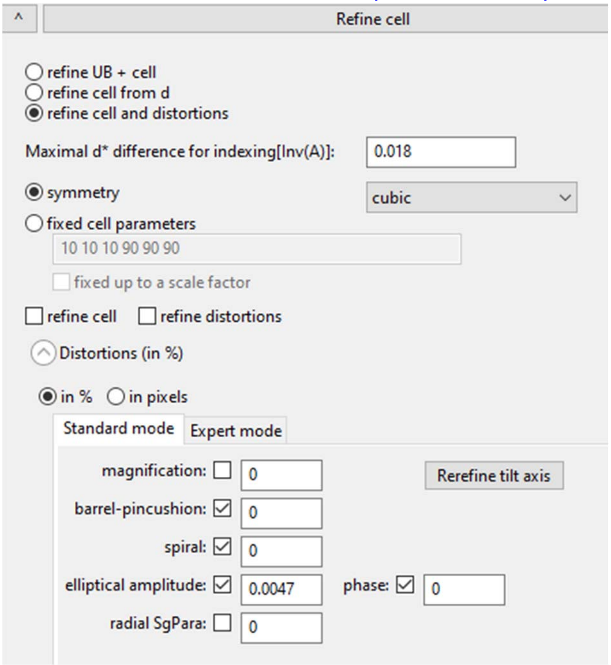


*It is generally not possible to refine freely elliptical distortion together with the lattice parameters because of strong correlation. This dataset is not an exception. Therefore, we use our knowledge about the symmetry of the unit cell and set symmetry to cubic.*

Click on “Refine cell” to proceed.

| | a | b | c | α | β | γ |
|---|---|---|---|---|---|---|
| cell: | 12.0018 | 12.0017 | 12.0017 | 90.000 | 90.000 | 90.000 |
| s.u.: | 0.0017 | 0.0000 | 0.0000 | 0.000 | 0.000 | 0.000 |

*You can see that the lattice parameter is far from the expected 11.9084 Å. There are two reasons for that: (i) the calibration constant was obtained for data without precession but precession with overexcited diffraction lens introduces image diminution, and (ii) the barrel-pincushion distortion did not converge to the correct value because it was influenced by the non-compensated effect of the parabolic distortion (radial SgPara coefficient).*

Click on “Finish”.

Open options for “Optimize reflection profile and geometry”.

Uncheck “refine global reflection profile parameters” and check “frame-by-frame optimization”. Uncheck “frame orientation angles” and “apparent mosaicity”.

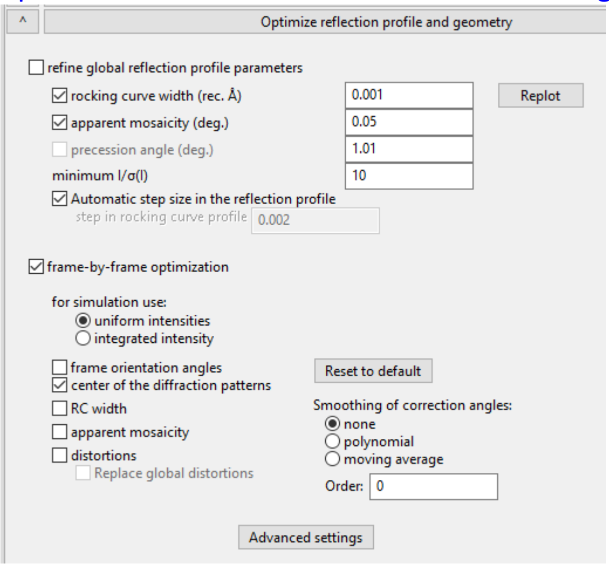


Click on “Optimize reflection profile and geometry” to proceed.

*This procedure generates simulated diffraction patterns, which can be compared with the experimental data.*

Switch to the “Image data” tab in the Main Panel, go to frame 28 (after the frame was simulated), set display cut off to 55, uncheck auto resize, set magnification to 110, choose “simulated” and “Combined images”.

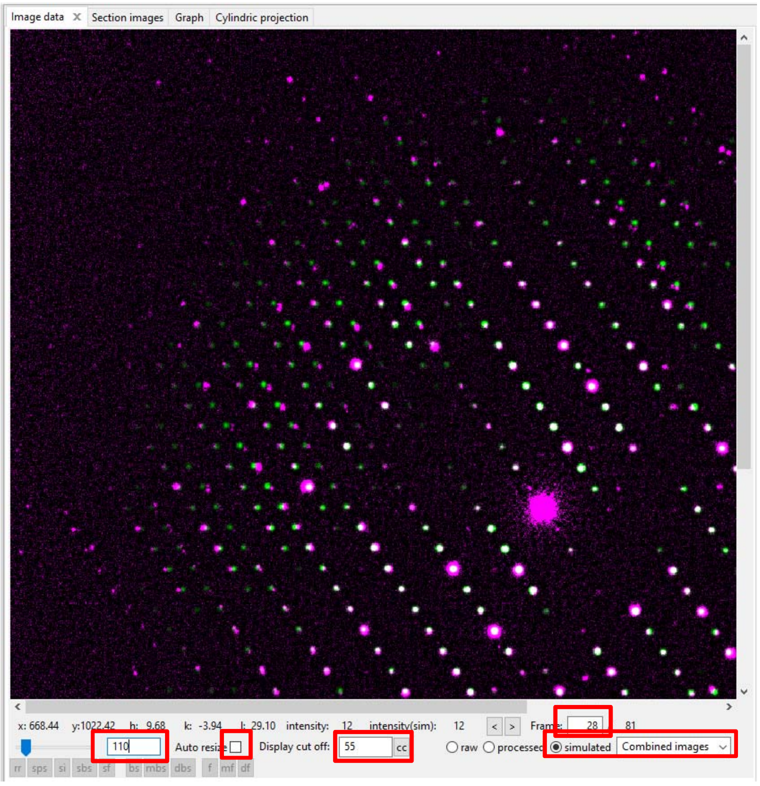


*Magenta and green represent the experimental and simulated data, respectively. In some places the reflections overlap nicely, however, in other parts (e.g. left part of the pattern) a significant shift can be observed.*

Run “Process frames for integration”.

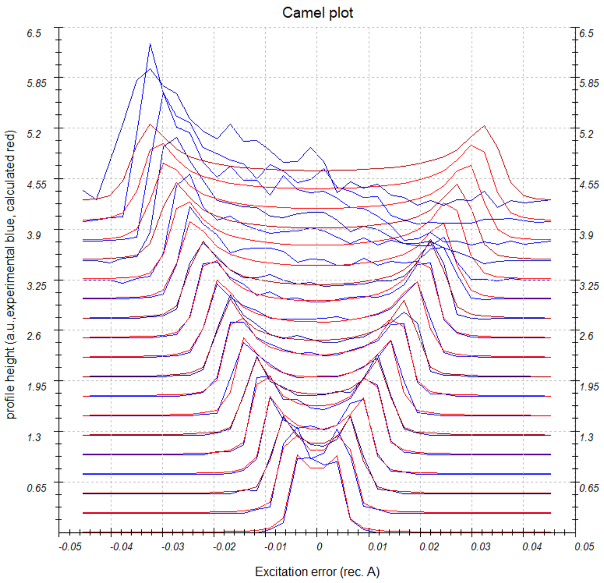


*Poor match between experimental and simulated diffraction patterns is also seen on the rocking curves and would result in incorrect integration of the intensities.*

Go to “Find unit cell and orientation matrix”, enable refinement of the “radial SgPara” coefficient.
Click “Refine cell” to proceed.

Click on “Rerefine tilt axis” after the cell refinement finishes.
Click “Refine cell” to final refinement.

| | a | b | c | α | β | γ |
|---|---|---|---|---|---|---|
| cell: | 11.9787 | 11.9787 | 11.9787 | 90.000 | 90.000 | 90.000 |
| s.u.: | 0.0009 | 0.0000 | 0.0000 | 0.000 | 0.000 | 0.000 |

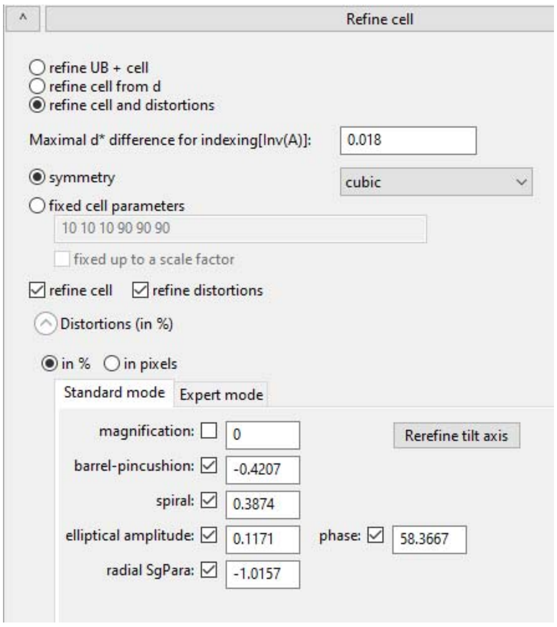


*All the distortions now converged close to the calibrated values. You can simulate the diffraction patterns and you will see that the simulation already fits very well to the experimental data. Before we do that, we refine also the magnification correction as the lattice parameter a = 11.9787 Å is still too large. The magnification correction due to the off-eucentric focus excitation of the diffraction lens in the precession data approximately corresponds to a half of the radial SgPara coefficient. We now determine the magnification correction exactly.*

Choose “fixed cell parameters” and write in the box 11.9084 11.9084 11.9084 90 90 90.
Enable refinement of the magnification correction.

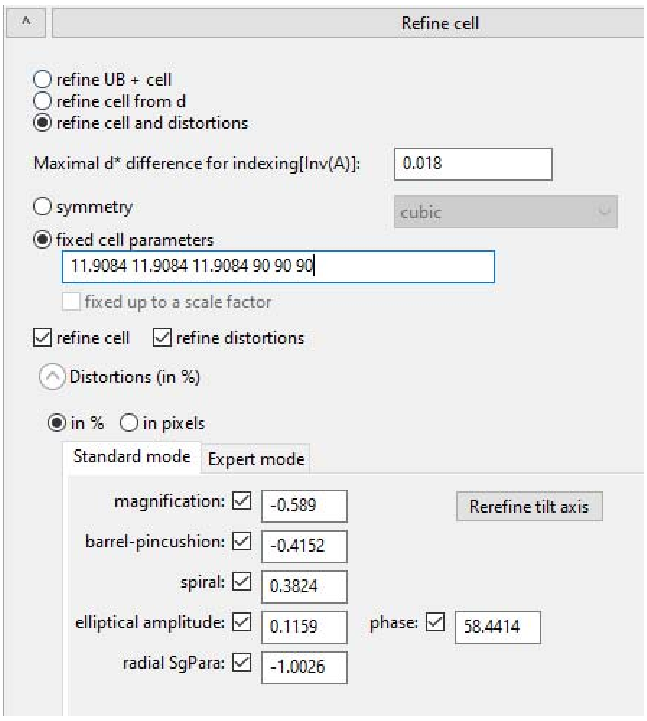


*The correct magnification correction refines to -0.589 %.*

Click on “Finish”.

Open options for “Optimize reflection profile and geometry”. Leave the setting as in the previous run. Run the optimization.

Switch to the “Image data”, go to frame 28 (after the frame was simulated), set display cut off to 55, uncheck auto resize, set magnification to 110, choose “simulated” and “Combined images”.

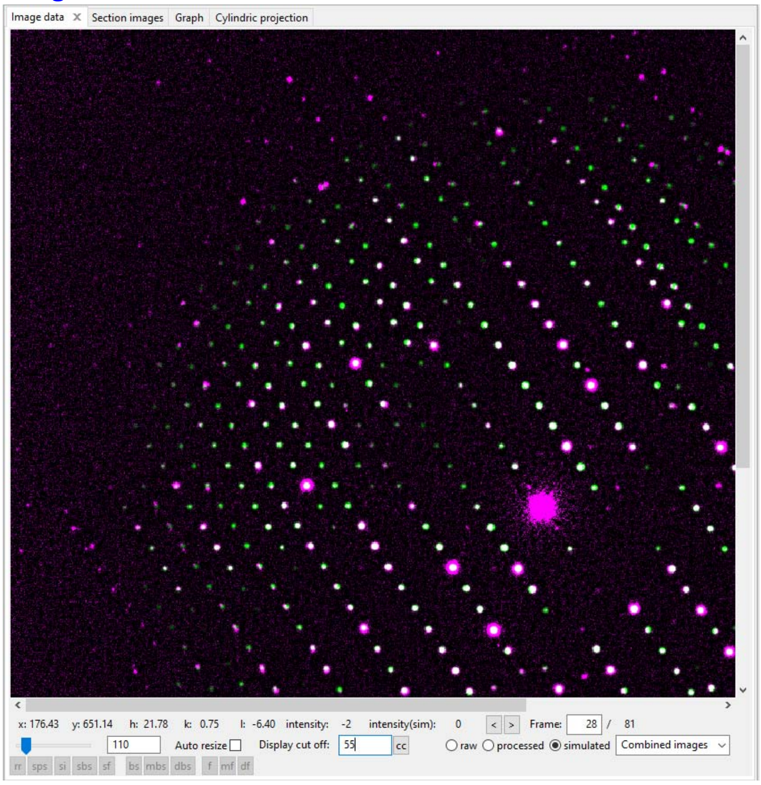

*You can see that now the simulated data (green) fit well to the experimental ones (magenta) with the exception of the reflection splitting in the experimental data, which is not accounted for in the simulated data.*

Run "Process frames for integration".

*The good match between experimental and simulated diffraction patterns is also seen on the rocking curves that correspond well and would result in correct integration of the intensities.*

*The integration of the intensities is now correct and the calculated profile fits well to the experimental rocking curves even at high resolution data.*

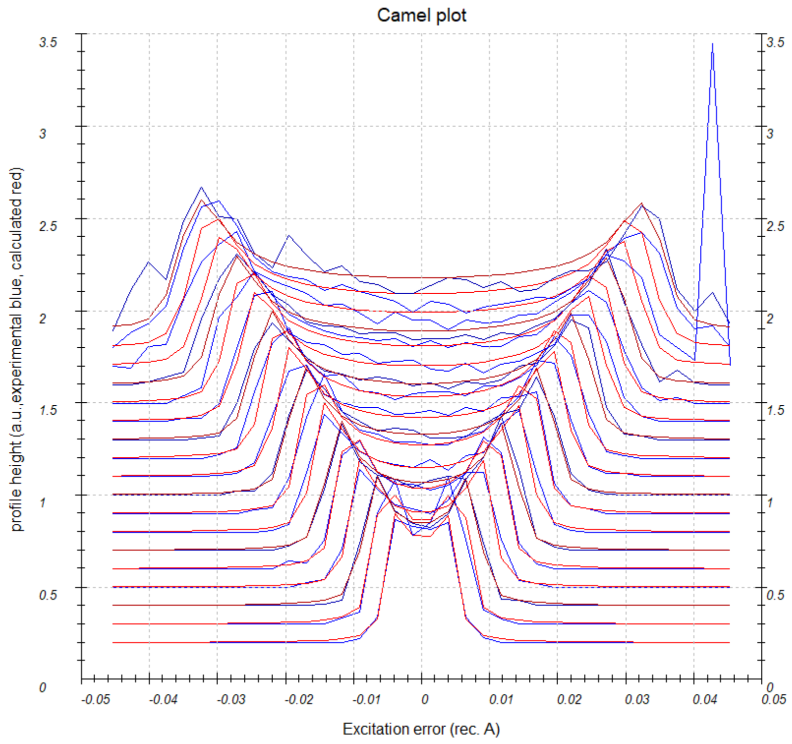

Supplement: Supplementary file 1 [file m-09-00735-sup1.pdf]
